# Standardization of Terminology, Definitions, and Outcome Criteria for Bleeding in Hereditary Hemorrhagic Telangiectasia: International Consensus Report

**DOI:** 10.1002/ajh.70011

**Published:** 2025-07-15

**Authors:** Hanny Al‐Samkari, Raj S. Kasthuri, Hans‐Jurgen Mager, Jenny Y. Zhou, Marcelo M. Serra, Bethany T. Samuelson‐Bannow, Layla N. Van Doren, Jay F. Piccirillo, Marianne S. Clancy, Keith R. McCrae, Sonia M. Thomas, Antoni Riera‐Mestre, Allyson M. Pishko, Sarah Sewaralthahab, James R. Gossage, Vivek N. Iyer, Cedric Hermans, Adrienne Hammill, Ingrid Winship, Meir Mei‐Zahav, Annette von Drygalski, Scott Olitsky, Marie E. Faughnan

**Affiliations:** ^1^ Massachusetts General Hospital Harvard Medical School Boston Massachusetts USA; ^2^ University of North Carolina‐Chapel Hill Chapel Hill North Carolina USA; ^3^ St. Antonius Hospital Nieuwegein the Netherlands; ^4^ University of California‐San Diego San Diego California USA; ^5^ Hospital Italiano de Buenos Aires Buenos Aires Argentina; ^6^ Oregon Health and Science University Portland Oregon USA; ^7^ Yale University New Haven Connecticut USA; ^8^ Washington University in St. Louis St. Louis Missouri USA; ^9^ Cure HHT Monkton Maryland USA; ^10^ Cleveland Clinic Cleveland Ohio USA; ^11^ Research Triangle Institute Durham North Carolina USA; ^12^ Hospital Universitari Bellvitge Barcelona Spain; ^13^ University of Pennsylvania Philadelphia Pennsylvania USA; ^14^ King Saud University Riyadh Saudi Arabia; ^15^ Augusta University Augusta Georgia USA; ^16^ Mayo Clinic Rochester Minnesota USA; ^17^ Catholic University of Louvain Brussels Belgium; ^18^ University of Cincinnati Cincinnati Ohio USA; ^19^ University of Melbourne Melbourne Australia; ^20^ Tel Aviv University Tel Aviv Israel; ^21^ University of Toronto Toronto Canada

**Keywords:** anemia, angiogenesis, bleeding, epistaxis, gastrointestinal bleeding, Hereditary hemorrhagic telangiectasia, HHT, iron deficiency, Osler‐Weber‐Rendu

## Abstract

Hereditary hemorrhagic telangiectasia (HHT, Osler‐Weber‐Rendu disease) is the second most common inherited bleeding disorder worldwide, affecting approximately 1 in 5000 people. Development of disease‐modifying and efficacious hemostatic agents to treat HHT has finally begun after decades without such medical therapies. However, the lack of consensus on standardized severity definitions, outcome criteria, and terminology remains a major obstacle to clinical investigation and therapeutic development in HHT. Additionally, with the ongoing repurposing of antiangiogenic agents and emerging development of novel HHT‐specific therapies, comparative clinical trials are expected in the future. Therefore, to end the problematic heterogeneity hindering these efforts, the Global Research and Medical Advisory Board (GRMAB) of the Cure HHT Foundation, an international group of recognized HHT experts, convened a conference of expert HHT clinician‐investigators from within GRMAB as well as invited external experts in HHT and bleeding disorders generally, patients with HHT, and patient advocates to define standard terminology and definitions for outcomes and severity classification for bleeding and anemia in HHT. These criteria and definitions should be adopted by regulators, investigators, and the pharmaceutical industry in the development and performance of interventional clinical trials and cohort studies to allow improved comparability between clinical trials, facilitate communication between clinicians and investigators, improve therapeutic guideline development, and provide a standardized framework for regulatory agencies.

**Search Strategy and Selection Criteria:**

Evidence for this report was systematically identified and evaluated utilizing two search strategies in Ovid MEDLINE, described in full detail in the Appendix, pp.4–13. The searches were conducted on January 7, 2025. The titles and abstracts of each record were reviewed, and the inclusion criteria were applied to all search results to identify full text articles to be retrieved for further review. All retrieved full texts were then reviewed to reach a final determination if a study met the inclusion criteria. Included references were then compiled into evidence tables, which were then utilized by the International Consensus Report Working Group throughout the development of report recommendations.

## 1 Introduction

Hereditary hemorrhagic telangiectasia (HHT, Osler‐Weber‐Rendu disease) is an inherited vascular bleeding disorder caused by dysregulated angiogenesis. HHT is inherited in an autosomal dominant fashion, with a prevalence of 1 in 5000 persons [1]. It is the second‐most‐common inherited bleeding disorder, behind only von Willebrand disease, and recent evidence suggests HHT may be the most morbid inherited bleeding disorder of women [2]. HHT is a multisystem disorder, but the most common manifestation is bleeding and its many downstream complications. Nearly all patients with HHT suffer recurrent epistaxis that may be severe and that frequently results in substantial medical and psychosocial morbidity [3, 4]. Approximately one‐third of patients with HHT suffer from clinically significant chronic gastrointestinal hemorrhage [5]. Recent evidence suggests high rates of heavy menstrual bleeding in women with HHT as well [6, 7], occurring in addition to the aforementioned bleeding manifestations. Any of these bleeding manifestations, alone or in combination, commonly result in iron deficiency or iron deficiency anemia, which may be severe and create a dependence on intravenous iron infusion and/or red cell transfusion [8, 9]. Arteriovenous malformations (AVMs) also occur in most patients with HHT, primarily in lung (~50%), liver (~70%), and brain (~10%–20%) [10]. These lesions result in additional complications, including but not limited to embolic stroke, pulmonary hemorrhage, intracranial hemorrhage, high‐output heart failure, brain abscess, and chronic liver disease. Despite the considerable morbidity experienced by patients from AVMs, patients and their caregivers highlight recurrent epistaxis as the most important disease manifestation [11]. Due to bleeding and AVM complications, studies have reported reduced overall survival in patients with HHT compared with the general population [12, 13].

Prior to the recent advances in the understanding of HHT pathophysiology and therapeutics that have transformed the HHT therapeutic landscape, management of HHT‐associated bleeding was primarily procedural, largely through the use of local ablative procedures and more drastic surgical operations (such as nasal closure operations for recurrent epistaxis) [14]. These treatments were largely studied retrospectively and published in the medical literature as case series [15]. However, the past decade has brought with it a paradigm shift in the management of bleeding in HHT from primarily procedural to primarily based in effective medical therapeutics [16, 17, 18, 19, 20, 21]. Along with this shift have come the first major randomized, placebo‐controlled clinical trials in HHT of antifibrinolytic and antiangiogenic drugs to manage bleeding [19, 22, 23, 24, 25, 26]. These pioneering studies and their promising results are responsible in large part for the paradigm shift in treatment and the emerging recognition from the pharmaceutical industry as well as government funding agencies of the serious unmet need in HHT [27]. However, they have also highlighted a key deficiency in the field: a lack of standardized terminology, definitions, and outcome criteria that has made study interpretation, comparison between studies, and development of new study protocols unnecessarily challenging. As long‐overdue therapeutic development in HHT accelerates and the field matures with large, randomized, pivotal clinical trials paving the way for the first drug approvals from regulatory agencies, the standardization of terminology, definitions, and outcome criteria is a necessity. Such standardization and elimination of heterogeneity is certain to improve evaluation of patient characteristics, reporting of clinical outcomes, and assessment of the clinical benefit of therapeutics. Therefore, the Global Research and Medical Advisory Board (GRMAB) of the Cure HHT Foundation, the largest international patient advocacy organization in HHT, convened an international group to develop the first global standardization consensus report on bleeding in HHT.

## 2 Methodology

The International Consensus Report (ICR) on the Standardization of Terminology, Definitions, and Outcome Criteria for Bleeding in Hereditary Hemorrhagic Telangiectasia was commissioned by the Cure HHT GRMAB in October 2024 at the 13th International Scientific Conference in Mandelieu‐La‐Napoule, France. While administrative support for the ICR was provided by Cure HHT, no monetary support of any kind was solicited from pharmaceutical companies or otherwise, and no member of the ICR Working Group or external reviewer received honoraria.

The ICR Working Group (expert panel), led by the chairperson (H.A.), included a total of 23 clinical and investigative HHT experts in HHT‐associated bleeding, bleeding disorders more generally, HHT clinical trial design/methodology, and biostatistics, as well as patient representatives and patient advocates, together from 9 countries across 5 continents, selected to ensure robust and diverse expertise, representation of the patient voice, and international scope. The goals, objectives, and topics of the document were determined and agreed upon during an in‐person GRMAB meeting and further refined by discussions with patient advocates. Through a prespecified systematic literature review process (Appendix, pp.4–6) of randomized clinical trials evaluating bleeding in HHT, 87 records were retrieved in full text for review and used to generate evidence tables for use by ICR Working Group members during the recommendation development process (Appendix, pp.7–13). These evidence tables were utilized to inform the development of all subsequent sections of this report. All ICR Working Group members completed disclosures that were reviewed by the chairperson and distributed to the entire ICR Working Group.

Ten members of the ICR Working Group formed the ICR Steering Committee, which was tasked with developing initial draft recommendations over a series of face‐to‐face virtual meetings. These draft recommendations were then circulated to the entirety of the ICR Working Group for intensive review and refinement and achievement of consensus. Rather than requiring all members to vote on all definitions and recommendations irrespective of their actual areas of expertise in this multidisciplinary disease, members of the ICR Working Group were encouraged to focus their efforts on their specific areas of expertise, and a voting process was not used. The draft report then underwent comprehensive external review by 15 international HHT bleeding and bleeding disorder topic experts as well as physicians in practice who care for patients with HHT who were not involved in ICR development, and their comments were collected and addressed (Appendix, pp.14–15). The final draft was ultimately circulated to all members of the Working Group, who gave explicit final approval (confirming agreement with the consensus document by all authors) prior to submission for publication.

## 3 Goals of Treatment

The aspirational goal of treatment for any disease is lasting cure. While a cure for HHT may be possible in the future thanks to ongoing advances in cellular and genetic therapies, currently available therapeutics and those in development focus on minimizing and/or preventing bleeding. The evaluation of these specific goals in clinical studies, and recommendations for proper clinical trial endpoints, can be found in Sections 5, 8.

### 3.1 Primary Treatment Goal

The expert panel agreed that the primary goal of treatment of HHT‐associated bleeding is the reduction of duration, frequency, and/or intensity of epistaxis as well as the reduction of gastrointestinal blood loss (in those with gastrointestinal bleeding).

### 3.2 Secondary Treatment Goals

The expert panel agreed that secondary goals of treatment of HHT‐associated bleeding (many of which are direct consequences of reducing epistaxis and gastrointestinal bleeding) include, in no particular order: (1) reduction of other sources of HHT‐associated bleeding (e.g., visceral and central nervous system AVM‐associated bleeding and skin and oral cavity bleeding); (2) reduction of iron deficiency anemia, non‐anemic iron deficiency, and requirements for hematologic support (intravenous iron infusions and red cell transfusions); (3) improvement of health‐related quality of life (HRQoL) and reduction of psychosocial morbidity; (4) reduction of healthcare utilization (e.g., emergency visits, hospital admissions, infusion center visits, hemostatic surgeries); (5) reduction of mortality; and (6) while doing all of the above, minimization of the toxicity of therapies for bleeding and the consequences of bleeding (including medical therapies, procedural therapies, hematologic support, and others; therapies with a high degree of known or anticipated toxicity should be evaluated only in severely bleeding patients). The expert panel agreed that, where possible, these impacts should be measured in studies (as defined in Sections 5, 8).

### 3.3 Treatment Goals Requiring Further Study

While anecdotal reports, as well as a recent large survey study, strongly suggested heavy menstrual bleeding complicating the course of approximately three‐quarters of women with HHT [6, 7], the scope and impact of heavy menstrual bleeding in HHT have yet to be rigorously defined. Given the relative ease of including menstrual bleeding measurement instruments in HHT bleeding trials (only minor added cost and participant burden) and the clear relevance to the topic, the expert panel recommends inclusion of exploratory endpoints evaluating menstrual bleeding in these studies.

Additionally, while the focus of this report and current drug development efforts are on the treatment of bleeding, it is recognized that disease‐modifying medical therapeutics that induce hemostasis via vascular remodeling and involution of telangiectasias may also have a therapeutic effect on both the treatment of existing visceral AVMs and their prevention [28, 29]. Given that the development of visceral AVMs occurs over years to decades, the expected timeline for observation of a therapeutic impact may be quite long. Therefore, the expert panel determined that inclusion of endpoints evaluating therapeutic impact on visceral AVMs in bleeding therapeutic trials is generally encouraged where feasible but should remain exploratory at this time. Measurement or sampling of skin, oral cavity, and nasal telangiectasias at baseline and tracking involution in response to therapies, primarily as a readily assessed marker of vascular remodeling, is also of interest as an exploratory endpoint but requires further study.

## 4 Bleeding Severity Classification and Definitions

Unlike hemophilia, which is characterized primarily by episodic bleeding that can be measured as an annualized bleeding rate, HHT is characterized primarily by ongoing chronic bleeding that may or may not be punctuated by episodes of acute, emergent, potentially life‐threatening bleeding. Therefore, classification of bleeding severity must account for the impacts of both chronic and emergent manifestations. Note that the expert panel is explicitly using the terminology “bleeding severity” rather than “disease severity” recognizing that the severity of bleeding complications and non‐bleeding visceral AVM complications may be disparate in individual persons. However, in people with no symptomatic visceral AVMs outside of the gastrointestinal tract, bleeding severity generally approximates overall disease severity. It is also important to note that bleeding in HHT is usually progressive over the lifespan, unlike many of its peer inherited bleeding disorders [4, 30]. An individual may progress from primarily mild bleeding to moderate bleeding and eventually severe bleeding over time. Additionally, environmental exposures (such as climate, allergies, diet, proper nasal moisturization or lack thereof) and therapeutic interventions may move an individual from one severity class to another.

### 4.1 Domains of Bleeding Severity

Recurrent epistaxis is present in over 95% of adults with HHT and is the uniting clinical manifestation in a disease with otherwise heterogeneous manifestations. Approximately one‐third of adults suffer from clinically significant chronic gastrointestinal bleeding, which is primarily manifest clinically by iron deficiency anemia and a requirement for hematologic support with intravenous iron and/or red cell transfusion. Bleeding manifestations may also result in significant psychological and HRQoL impacts. Given this, the expert panel agreed that categorizing bleeding severity in HHT requires evaluation of three specific domains: (1) measurement of epistaxis severity (severity includes frequency, duration, and intensity) with a validated instrument or epistaxis diary, (2) hematologic support requirements to achieve and maintain a normal hemoglobin and normal iron stores, and (3) the individual report of bleeding impact. All three domains should be assessed to appropriately categorize a patient's bleeding severity in clinical practice. However, to promote objectivity and consistency across clinical studies and HHT populations, until more data on the use of HHT‐specific HRQoL measurement scales (Section 7) is available, only the first two domains can be used for severity classification in clinical studies (Table 1). The expert panel recommends that severity classification in clinical studies be done at baseline, primarily for purposes of eligibility and stratification, and not used as an endpoint (appropriate endpoints are discussed in Sections 6, 8). Critically, the expert panel emphasized that this severity classification must not be used by health systems, insurance companies, or other payors to deny care to patients prescribed a therapy.

**TABLE 1:** Bleeding Severity Classification.

Classification	Domain 1: epistaxis severity measurement^a^ with validated instrument or epistaxis diary	Domain 2: hematologic support requirements to maintain normal hemoglobin and normal iron stores^b^	Domain 3: individual report of bleeding impact^c^
Mild	ESS < 4.00 NOSE HHT average score < 1.00	Dietary iron or oral iron supplementation alone	Minimal reported limitations in work, school, or interpersonal relationships
Moderate	ESS 4.01–7.00 NOSE HHT average score 1.01–2.00 Monthly^e^ frequency of ≥ 20 episodes plus monthly duration^d^ of ≥ 80 min (cumulative)	IV iron infusion totaling < 2000 mg elemental iron in the previous year^e^ Combined IV iron and red cell transfusion requirement of < 8 RUEs^f^ in previous year^e^	Modest limitations in instrumental activities of daily living Limitations in work or school (e.g., reduced hours, altered role, inability to pursue desired extracurricular activities) causing emotional, social, or financial distress Limitations in interpersonal relationships (e.g., social interactions, romantic relationships, public activities) causing emotional, social, or financial distress
Severe	ESS 7.01–10.00 NOSE HHT average score > 2.00 Monthly^e^ frequency of ≥ 30 episodes plus monthly duration^d^ of ≥ 200 min (cumulative)	IV iron infusion totaling ≥ 2000 mg elemental iron in the previous year^e^ Combined IV iron and red cell transfusion requirement of ≥ 8 RUEs^f^ in previous year^e^ Maintains subnormal hemoglobin while IV iron dependent but does not require red cell transfusions^g^	Significant limitations in instrumental activities of daily living or any limitations in basic activities of daily living Unemployment (voluntary or otherwise) Inability to attend school Results in a mood disorder (e.g., major depressive disorder), anxiety disorder (e.g., generalized anxiety disorder, agoraphobia, social phobia, panic disorder, post‐traumatic stress disorder) or other mental health disorder, as diagnosed and attributed primarily to HHT bleeding by a mental health professional; the psychiatric diagnosis must be currently active to qualify (rather than only historical)
Very Severe	—	Regular red cell transfusion (red cell transfusion dependence, see Table 4) required to maintain acceptable (i.e., safe) hemoglobin^h^	—

*Note*: The overall bleeding severity classification equals the most severe category measured in any single domain. Meeting a single criterion (bullet point) within the severity classification of a domain is sufficient for assigning that severity classification (all criteria do not need to be met). All three domains may be assessed in clinical practice for determination of bleeding severity. However, only domains 1 and 2 should be used for classification in clinical studies. Justifications for given cutoffs for epistaxis frequency and duration (domain 1) and IV iron (domain 2) are provided in the Appendix, pp.22–23. The Epistaxis Severity Score is a 6‐question instrument scored between 0.00 and 10.00 and the Nasal Outcome Score in HHT is a 29‐item instrument scored between 0.00 and 4.00. In both cases, higher numbers indicate more severe epistaxis. Free online calculators for both scores are available (ESS, https://curehht.org/resource/epistaxis‐severity‐score/and NOSE HHT, https://outcomesresearch.github.io/nose‐hht/). ESS, Epistaxis Severity Score; NOSE HHT, Nasal Outcome Score for Epistaxis in HHT; IV, intravenous; RUE, red‐cell unit equivalent.

^a^ Should be performed over a reference time frame of at least 4 weeks for baseline severity classification in clinical studies.

^b^ A normal hemoglobin is defined as a hemoglobin value at or above the lower limit of normal according to local laboratory ranges. Normal iron stores are defined as a ferritin ≥ 50 ng/mL (ideally with a transferrin saturation ≥ 20%, though a transferrin saturation ≥ 20% is not required for classification purposes) [14, 31]. Note that ferritin may be elevated by inflammation, and transferrin saturation must be evaluated while fasting. Domain 2 classifications presume proper hematologic support administration in individuals with HHT, according to the guidelines. If a patient on oral iron has a normal hemoglobin but a ferritin persistently < 50 ng/mL despite optimization of oral iron, this individual requires intravenous iron to achieve normal iron stores (which should be completed without delay, including prior to clinical trial enrollment) and would therefore be classified as “Moderate” in domain 2 for the purposes of severity classification.

^c^ Manifestations listed must be specifically and primarily relatable to epistaxis or other HHT‐specific bleeding, or consequences of bleeding (such as anemia) in order to meet listed criteria.

^d^ Measurement of duration may be impacted by nasal packing and interventions, including self‐packing employed by some individuals. In general, the expectation is that people will make attempts to stop epistaxis, and the duration should represent the time from initiation to the time of complete cessation of bleeding for a given episode.

^e^ “Monthly” is defined as 4 weeks (28 days). The “previous year” is defined as a full calendar year (e.g., if evaluating on November 2, 2026, the evaluation period is inclusive of November 3, 2025 through November 2, 2026).

^f^ One red‐cell unit equivalent (RUE) equals 1 unit of packed red cells or 250 mg elemental iron, as described in detail in the Appendix, p. 24.

^g^ An individual who maintains a hemoglobin less than 12 g/dL while receiving ≥ 1,000 mg intravenous elemental iron monthly.

^h^ There is no specific IV iron administration threshold after which an individual is deemed to begin requiring regular red cell transfusions; this occurs when bleeding is chronically brisk enough that the bone marrow cannot keep up with blood loss despite abundant elemental iron. However, in general, these individuals will not maintain a hemoglobin above 9 g/dL despite receiving 1000 mg or more of intravenous elemental iron monthly. Individuals defined as being red cell transfusion dependent (requiring “regular red cell transfusion” to maintain an acceptable hemoglobin) will usually require transfusion at least once weekly but will require a minimum of 2 units of red cells per month to maintain an acceptable hemoglobin (Table 4).

### 4.2 Validated Epistaxis Severity Measurement

The expert panel agreed that epistaxis severity measurement should be performed with one or more validated, HHT‐specific epistaxis severity instruments that assess epistaxis severity retrospectively and/or measured prospectively using an epistaxis diary (electronic epistaxis tracker app or paper diary), Table 1. The instruments currently highlighted for use are the Epistaxis Severity Score (ESS) [32], a validated instrument used for evaluation of epistaxis severity in HHT (assessed over a reference time frame of 1–3 months; initially validated for 3 months), or the Nasal Outcome Score for Epistaxis in HHT (NOSE HHT) [33], a more comprehensive instrument assessing epistaxis over a reference time frame of 2 weeks. Both instruments assess frequency, duration, and intensity of epistaxis and have simple numeric ranges and scoring that define mild, moderate, and severe epistaxis (additional details on each instrument can be found in the Appendix, pp.16–21). By nature, as patient‐reported outcome measures, these instruments are at least partially subjective. There are several important nuances in scoring the ESS, and best practices must be followed (as detailed in Table 2). In the setting of prospective studies, measurement of epistaxis frequency and duration using an epistaxis diary can supplement or substitute for an instrument‐based measurement in defining domain 1 epistaxis severity, an approach that may be a more precise measurement of overall epistaxis severity [19, 24, 34]. The expert panel recognizes that other epistaxis severity instruments have been developed for use in HHT and that additional instruments will be developed in the future that may supplant the ESS and NOSE HHT and does not object to the use of alternative instruments in place of these, so long as they are appropriately validated. Given the potential week‐to‐week variability in epistaxis in a person with HHT, the expert panel recommends a reference time frame of evaluation of at least 4 weeks for baseline severity classification in clinical studies.

**TABLE 2:** Recommendations for measuring epistaxis in clinical trials.

Modality	Recommendations	Notes
Epistaxis Diary	Electronic diaries should be used over paper diaries whenever possible/feasible (particularly for large, pivotal trials, industry‐sponsored trials, and registrational trials), but paper diaries may be used when use of an electronic diary is not possible or feasible An electronic diary with regular secure cloud upload should be used, when possible, to minimize potential for lost data (which is greater with paper diaries or data stored locally on a device) Electronic diary should have a data entry lockout of 3–5 days to balance encouragement of timely data entry, allow for correction of entry errors, and minimize potential for recall bias Nosebleeds should be defined as blood coming out of the nose or down the back of the throat; blood‐tinged mucus, for example with nose‐blowing or a sneeze, is not a nosebleed Diary should assess epistaxis frequency, duration, and intensity (flow intensity) as separate domains recorded by the participant, ideally recording nosebleeds as discrete events with a specific duration and flow intensity assigned to each event ○ Participants should enter bleed duration as a continuous variable (e.g., 17 min), not as a categorical variable (e.g., 16–30 min) to maximize data granularity and quality ○ Flow intensity is ideally assessed utilizing descriptive categories (e.g., dripping, steady stream, pouring, gushing) or a visual analog scale ○ Additionally, the diary should include a binary option for each bleed specifying if a bleed was spontaneous or provoked by a specific physical action (e.g., blowing one's nose, bending over, sneezing, etc.) Participants should be educated on what is considered a long nosebleed with occasional brief stops in flow versus multiple nosebleeds that should be recorded as separate events ○ The expert panel recommends that nosebleeds that stop completely for 30 or more minutes before bleeding resumes should be considered separate nosebleeds and recorded as separate entries in the diary Participants who self‐pack to stop nosebleeds should be instructed to be consistent in how they enter their nosebleed duration and intensity throughout the entire duration of a study, and it is recommended that the end of a nosebleed be consistently defined as cessation of bleeding, whether or not packing is used Electronic diary apps should prompt the participant to enter data at least once daily, providing a simple and user‐friendly interface A secure web portal should be available to enter daily epistaxis data without gaps in the event that the personal device used for entry is lost or malfunctioning; the web portal must have all of the functionality of the app and should have a similar interface If no nosebleeds were recorded during the preceding day, the app should prompt the participant to confirm that they did not have nosebleeds for the entirety of that preceding day A third‐party monitor should remotely monitor participant adherence with an electronic diary and provide regular reports to the study team; either the monitor or a member of the study team should reach out directly to participants who are nonadherent for a period of 3–5 days (depending on the duration of the lockout period; participants should be contacted to enter data before lockout occurs) Participant training on the electronic diary app should occur for a period of 1–4 weeks during the screening period, prior to the collection of study‐relevant data Diaries should include a means to designate “special circumstance bleeds,” that should be collected differently than standard bleeds, including: ○ Epistaxis episodes occurring during sleep: In general, participants should not be asked to answer detailed questions regarding duration and flow intensity as this is not possible (simple assessments of severity, e.g., a “minor” or “major” bleed, based on the participant's assessment of the amount of blood lost, are appropriate). If these questions are asked, the study should have a prespecified process for how this data is to be used given that recorded duration, for example, may not be reliable ○ Posterior‐only epistaxis episodes: For posterior‐only nosebleeds, actual confirmation of bleeding such as the taste of blood or spitting blood is required for that to count as a bleed; if the participant only feels posterior drainage without confirming blood, it should not be counted Because of the participant burden of a daily diary, continuous daily use of the diary is ideally required only during the baseline severity assessment period (if epistaxis diary parameters are being used to determine severity category/eligibility) and the main study period during which the primary and secondary endpoints relating to epistaxis diary data are being adjudicated. If it is to be continued in an open label extension, consider limiting the total duration of continuous daily use to 12 months (including double‐blind phase and extension), or requiring only intermittent use (e.g., one month every three months), although the latter can introduce challenges associated with halting and restarting the use of the diary. In general, it is not feasible to require all participants to reliably fill out a daily epistaxis diary consistently and indefinitely for years during an open label extension	Recommendations developed based on clinical trial experiences including PATH‐HHT trial, EPICURE trial, VAD044002 trial, and others
Epistaxis Severity Score	ESS should be investigator‐administered during a live encounter (e.g., in person, telephone, videoconference) with the participant, with confirmation of comprehension and accurate reporting ○ Investigators should be explicitly trained on proper use of the ESS (generally by the sponsor) prior to enrolling participants ○ If not administered by an investigator, it is acceptable for a research coordinator to administer the ESS, but they should be explicitly trained on proper use of the ESS (generally by the sponsor) and initially supervised by an investigator before independently administering the ESS to participants Ideally, the ESS should be administered to a given participant by the same investigator, where feasible. Where not feasible, the number of different individuals administering the ESS to the same participant over the course of a study should be minimized The reference time frame for the ESS should be no less than 1 month (4 weeks) and no longer than 3 months (12 weeks) The study protocol should explicitly define all vague aspects of ESS items to ensure conformity among sites and investigators in interpreting and answering items ○ Item 4 asks, “Have you sought medical attention for your nose bleeding?” The term “sought medical attention” should be defined in the protocol (e.g., “‘sought medical attention’ includes physical visits to a clinic or emergency department, but does not include a phone call to the clinic”). ○ Item 6 asks, “Have you received a red blood cell transfusion SPECIFICALLY for nose bleeding?” This is challenging to adjudicate in participants with concomitant epistaxis and gastrointestinal bleeding. In general, the expert panel recommends adjudicating this question as “yes” if the participant has had a transfusion and has at least moderate severity epistaxis (even if they have significant gastrointestinal bleeding), to minimize subjectivity. Of note, receipt of intravenous iron only should not trigger a “yes” to this question, and participants may mistake iron for blood (and vice versa). Therefore, records should always be verified to confirm what hematologic support has been given “Anemic (low blood counts)” as per item 5 should be objectively defined (e.g., as a hemoglobin value, not a subjective question) and consistently applied (e.g., “a hemoglobin value below the laboratory reference range performed within 7 days of the ESS assessment”) ○ The expert panel recommends against the use of race‐based hemoglobin reference ranges ○ The expert panel acknowledges ongoing controversy regarding sex‐based laboratory reference ranges; in general, caution should be exercised to avoid use of inappropriately low hemoglobin lower limits of normal for women The investigator or coordinator administering the ESS should record the response for each of the six items in the medical record, which is then transferred to a case report form. A calculator can be used on the form to calculate the total score; it is not sufficient to record only the total score in the medical record without including the response for each item.	The ESS was originally validated for a 3‐month (12‐week) reference time frame; however, it has been successfully used as an efficacy endpoint in major trials (e.g., PATH‐HHT) with a 1‐month (4‐week) reference time frame, and so use in this manner is considered acceptable In general, shorter reference time frames are expected to result in less recall bias
Nasal Outcome Score for HHT	NOSE HHT must be administered with a 2‐week reference time frame NOSE HHT should be completed in print or electronic format by the participant Assessment with this instrument over a period longer than 2 weeks requires interval measurements every 2 weeks (with averaging of the score obtained at each measurement to obtain an average NOSE HHT score over the assessed interval)	The requirement to administer NOSE HHT every 2 weeks may result in less recall bias with this instrument than with the ESS, though this has not yet been evaluated

Abbreviations: ESS, Epistaxis Severity Score; NOSE HHT, Nasal Outcome Score for Epistaxis in HHT.

### 4.3 Hematologic Support Requirements to Maintain a Normal Hemoglobin and Normal Iron Stores

Restoration and maintenance of a normal hemoglobin and normal iron stores with adequate iron supplementation is the standard of care in HHT and a crucial pillar of best practice in HHT management [15, 35]. Table 1 lists criteria recommended by the expert panel to define bleeding severity based on hematologic support requirements. Note that without concurrent bone marrow pathology, intravenous iron effectively functions as a delayed onset red‐cell transfusion in individuals with HHT. For this reason, and because acute bleeds also occur that necessitate intermittent red‐cell transfusion, the expert panel recommends evaluation of hematologic support requirements using red‐cell unit equivalents (RUEs) in order to account for both support modalities in a single unit of measurement (1 RUE equals 1 unit of red cells or 250 mg elemental iron; additional detail is in the Appendix, p.24) [36]. Of note, the panel recognized the potential confounding nature of menstrual blood loss (which, when heavy, may or may not be a consequence of HHT) in measuring hematologic support requirements in menstruating persons. Finally, the expert panel included the special category of very severe for individuals requiring regular red‐cell transfusions (usually required at least once weekly) to maintain an acceptable hemoglobin because of the uniquely challenging clinical circumstances of this group. The exact prevalence of this subgroup is not known but is thought to represent fewer than 5%–10% of people with HHT who receive proper intravenous iron management.

### 4.4 Individual Report of Bleeding Impact

While its relative subjectivity limits its applicability to severity classification in clinical trials, the individual report of bleeding impact (Table 1) is an essential component of severity evaluation in clinical practice. Note that the circumstances listed in Table 1 are an extensive but not an exhaustive list.

### 4.5 Major Acute Bleeding

While most bleeding in HHT is chronic, in recognition of the seriousness and life‐threatening potential of acute bleeds, the expert panel defined a major acute bleed in HHT by adapting the definition for major bleeding from the International Society on Thrombosis and Haemostasis [37] to best reflect the HHT disease state. A major acute bleed in HHT is defined as satisfying one or more of the following criteria: (1) acute‐onset severe epistaxis or gastrointestinal bleeding necessitating urgent intervention performed by a healthcare provider (e.g., nasal packing, administration of intravenous antifibrinolytic, or ablative procedure); (2) pulmonary, central nervous system, or other solid organ bleeding due to a ruptured AVM; (3) acute bleeding that results in a drop in hemoglobin of ≥ 2 g/dL over a 24‐h period; or (4) acute bleeding that results in hemodynamic instability or a requirement for packed red cell transfusion (in excess of the individual's baseline maintenance red cell transfusion requirements, if any).

## 5 Epistaxis Measures and Endpoints

Clinical studies evaluating bleeding in HHT should broadly include epistaxis‐based endpoints, hematologic endpoints, and in prospective studies, HRQoL endpoints. However, the relative ranking of these endpoints depends on the bleeding severity of the population under study.

Because recurrent epistaxis is present in nearly all people with HHT and other bleeding and hematologic manifestations are more variably present, the expert panel agrees that epistaxis‐based endpoints are most appropriate for use as the primary endpoint in clinical trials of participants with moderate to severe HHT‐associated bleeding. The expert panel recommends that in general, pivotal clinical trials of therapeutics in HHT should enroll participants with moderate and severe bleeding (Section 4) to evaluate the therapeutic in the broadest population for which treatment is both indicated *and* response can be best measured, and utilize an epistaxis‐based primary endpoint. This approach has been successful in large, randomized controlled trials in HHT demonstrating the efficacy of tranexamic acid, pomalidomide, and VAD044 [19, 24, 25]. The inclusion of participants with moderate bleeding will, in most cases, result in an inability to feasibly power a trial using a hematologic primary endpoint (see Section 6 and Appendix, pp.7–9). Agents with a high anticipated toxicity burden may be more appropriate only in participants with severe bleeding, and in this case, a hematologic primary endpoint may be optimal (see Section 6). Epistaxis‐based primary endpoints should assess epistaxis severity utilizing either an epistaxis diary or a validated epistaxis severity instrument. If an instrument is used, the expert panel highlights the ESS and NOSE HHT because these instruments are validated; other similarly validated instruments may be considered as well if best practice principles of consistency and objectivity are applied with their use in a research setting. While the ESS has been utilized successfully in several clinical studies (Table S1), relatively modest changes in reporting by the patient can result in notable shifts in scoring, and several members of the expert panel expressed concerns about its suitability as a primary endpoint [38]. Recommendations regarding how these tools should be applied to collect data are detailed in Table 2. When utilizing an instrument developed in one language in a different language, the instrument should be translated by a professional translation service according to the standards of the industry [39].

A placebo effect has been consistently observed with ESS, NOSE HHT, and epistaxis diary‐based endpoints in HHT trials [19, 22, 23, 24, 25, 40]. The expert panel has noted that based on the available data, this effect appears to be more pronounced with recall‐based instruments such as the ESS as opposed to epistaxis diary‐based endpoints (e.g., epistaxis duration). Moreover, electronic epistaxis diaries with built‐in data entry lockout after a reasonable length of time (e.g., 3–5 days) likely reduce epistaxis diary recall bias relative to paper diaries which can be filled in at any time between visits. This reduction in recall bias may minimize the placebo effect [22, 24]. Though more operationally challenging and more burdensome to the participant, a well‐implemented epistaxis diary is likely superior to the use of a recall‐based instrument as a primary endpoint. Whenever one is selected as a primary endpoint, however, the expert panel recommends inclusion of the other as a secondary efficacy endpoint.

## 6 Hematologic Measures and Endpoints

Hematologic endpoints must be approached with caution in HHT clinical trials. While attractive because of their objectivity, the principal hematologic endpoints of hemoglobin, elemental iron infused, and red‐cell units transfused are three dynamic components of the same measurement (rate of iron/red cell flux through the body) in the chronically bleeding person with HHT [36]. The designation of one of these three alone as an endpoint is likely to result in incomplete conclusions that may be misleading [23, 26]. For example, a participant with moderate bleeding enrolls in a trial with a hemoglobin of 14.2 g/dL, appropriately normal owing to 750 mg elemental iron infused (3 RUEs) in the preceding 6 months. The participant completes the trial, and his final hemoglobin is measured at 14.1 g/dL, but his bleeding substantially improved, and he required only 250 mg of elemental iron infused (1 RUE) during the 6‐month trial period, a 66% reduction in hematologic support requirements while maintaining a stable hemoglobin. A hemoglobin endpoint would conclude no improvement in this participant, when in actuality he achieved marked improvement in bleeding. Additional examples of misleading results with the use of solitary hematologic endpoints are given in the Appendix, pp. 25.

**FIGURE 1:**
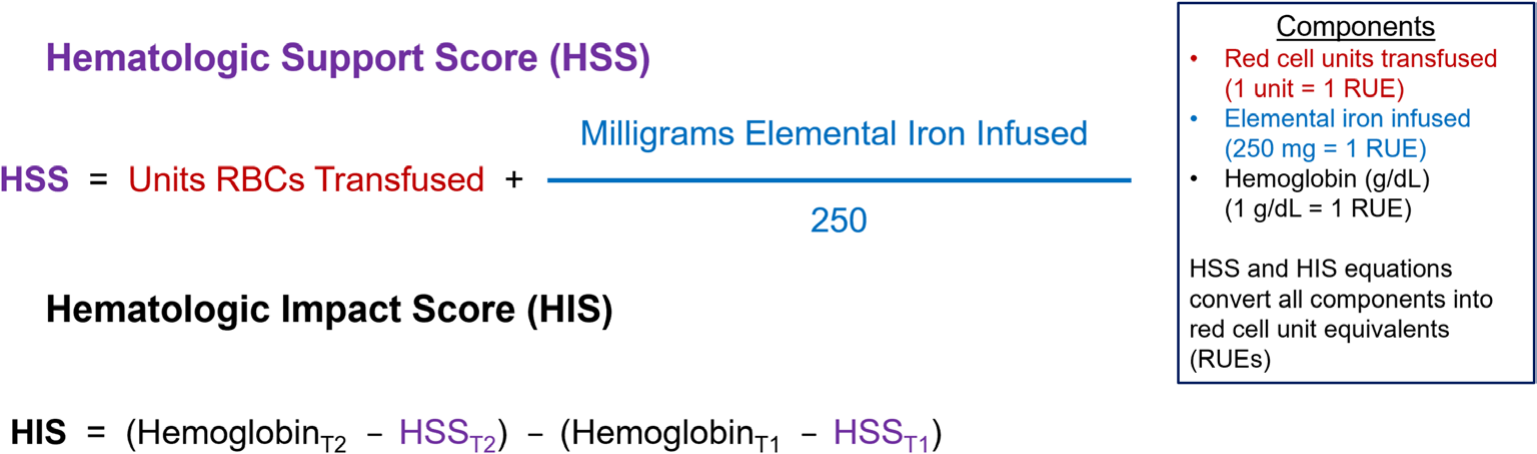
Composite Hematologic Endpoints, the Hematologic Support Score (HSS) and Hematologic Impact Score (HIS). The HSS measures the total amount of hematologic support over a specified time frame (e.g., 3‐month HSS; any time frame may be used, but a minimum of 3 months/12 weeks is recommended in clinical studies); a higher number signifies more required hematologic support and worse overall bleeding. The HIS assesses the total hematologic impact over a reference time frame by integrating the change in hemoglobin and the change in the HSS over that period of time; a higher number signifies a greater improvement in hematologic status over the time period. Example: A person with HHT receives 2 units RBCs and 1000 mg elemental iron during a 6‐month period (T1), and at the end of the period her hemoglobin is 10.5 g/dL (Hemoglobin at T1). The 6‐month HSS for this person is 6.00 red‐cell unit equivalents, or RUEs (HSS at T1). She then enters a 6‐month interventional clinical trial, during which time she receives a novel therapeutic to treat bleeding. During the 6‐month trial period, she receives 0 units RBCs and 500 mg elemental iron, and at the end of the period her hemoglobin is 11.7 g/dL (Hemoglobin at T2). Her post‐intervention 6‐month HSS is 2.00 RUEs (HSS at T2). Her HSS improved by 67%, and her HIS is +5.2 RUEs, representative of an improvement in overall hematologic status of 5.2 RUEs during the 6 months receiving treatment on trial compared with the 6 months prior. Both the HSS and HIS demonstrate a substantial improvement in her hematologic status achieved through reduced bleeding (and a much greater improvement recognized than had a solitary hemoglobin endpoint been used). Of note, these composite endpoints must be adjusted for use in children, as red cell transfusion quantities in children are weight‐based and not given in standardized units. T1 = Time 1 (baseline), T2 = Time 2 (end). [Color figure can be viewed at wileyonlinelibrary.com]

For this reason, the expert panel recommends use of composite hematologic endpoints in clinical trials in HHT, such as the hematologic support score (HSS), which combines iron infusion and red cell transfusion (a higher number signifies greater need for hematologic support during a reference time frame), or the hematologic impact score (HIS), which assesses the total hematologic impact over a reference time frame by accounting for both the change in hemoglobin and the change in the HSS over that period of time (a higher number signifies a greater improvement in hematologic status over the time period) (Figure 1) [36]. Both scores measure these components over a specified time frame and report out a simple numeric value in RUEs. The panel makes this recommendation with the acknowledgment that with few exceptions (e.g., the TrUST‐HHT study [NCT04404881], which is ongoing), this approach is relatively novel, and further study will be required to confirm its validity. Nevertheless, the serious pitfalls of relying upon solitary hematologic endpoints in HHT trials are clear [23, 26]. Until composite measures are well‐validated, the expert panel recommends additional inclusion of the solitary hematologic endpoints of hemoglobin concentration, elemental iron infused, and red‐cell units transfused as either non‐key secondary or exploratory endpoints.

Consistent with the expert panel's recommendation above to include participants with moderate and severe bleeding in pivotal clinical trials of agents to treat HHT‐associated bleeding, the expert panel recommends caution with the use of a hematologic endpoint (solitary or composite) as a primary or co‐primary endpoint in such trials. The panel makes this recommendation given the substantial heterogeneity in baseline hematologic support requirements among participants with moderate bleeding, resulting in a high risk of randomization failure between study groups and challenges properly powering such a trial (Appendix, pp.7–9). Conversely, for trials limiting the studied population to those with severe bleeding, a composite hematologic endpoint such as the HSS is an ideal primary endpoint. The severe bleeding population has sufficiently high baseline hematologic support requirements to adequately power such a trial and is typically enriched with participants suffering from chronic gastrointestinal bleeding who have high baseline hematologic support requirements. Evaluation of chronic gastrointestinal bleeding remains reliant on hematologic measurements (ideally composite hematologic endpoints) as there is currently no reliable means to quantify gastrointestinal blood loss specifically or to separate it from concurrent epistaxis blood loss.

Additionally, the expert panel recommends the use of protocolized thresholds for the administration of hematologic support in prospective HHT clinical trials. Practice variation between investigators, countries, and hospitals introduces a major source of potential confounding and bias pertaining to the administration of red cell transfusions and intravenous iron and may cause a failure to accurately assess the therapeutic potential of a drug [41]. Protocolized ferritin and transferrin saturation thresholds (e.g., ferritin < 50 ng/mL and/or transferrin saturation < 20%) [31] should be utilized to trigger iron infusion, which should require infusion of a narrow range of iron quantity (e.g., 400–600 mg or 800–1000 mg) for each occurrence. A narrow range of iron quantity is crucial in allowing for necessary flexibility to use one of many different IV iron formulations while controlling the amount of iron administered with each triggered infusion. Similarly, explicit hemoglobin transfusion thresholds should be defined in the protocol to trigger transfusion of a certain number of red‐cell units (e.g., hemoglobin < 8.5 g/L triggers transfusion of 1 unit, hemoglobin < 7.5 g/dL triggers transfusion of 2 units, etc.). For red‐cell transfusion‐dependent participants, investigators may choose to make transfusion thresholds individualized to a given participant, if they desire, as has been done successfully in other trials enrolling participants with transfusion‐dependent anemia [42].

As mentioned previously, heavy menstrual bleeding may be more prevalent in HHT than in the general population and could serve as a confounder in the accurate assessment of hematologic endpoints in menstruating women. The expert panel recommends that clinical trial protocols limit the introduction of medications or interventions that may significantly impact menstrual flow (e.g., intrauterine contraceptive placement, initiation of oral contraceptives or endometrial ablation), unless medically necessary, during a clinical trial in which hematologic endpoints are designated as primary or key secondary endpoints in order to best keep menstrual blood loss stable in the absence of benefit induced by the investigational therapy. Additionally, in such trials, the expert panel recommends that a method to understand the evolution of menstrual blood loss while hematologic endpoints are being evaluated be used, such as the Pictorial Blood Assessment Chart (PBAC) questionnaire and/or the Menstrual Bleeding Questionnaire (MBQ) [43, 44]. Under no circumstance should these additional recommendations discourage or reduce the participation of women in HHT clinical trials.

Lastly, when hematologic endpoints are designated primary or key secondary, the panel recommends that clinical trials impose a requirement for either discontinuation of oral iron prior to enrollment (and sole management with IV iron) or for oral iron supplementation to remain stable during all periods that hematologic endpoints are measured, to eliminate the potential for confounding on the results of hematologic endpoint measurements as it is impossible to determine the amount of iron absorbed from the gut of each individual participant taking oral iron.

## 7 Health‐Related Quality of Life Measures and Endpoints

HRQoL, an individual or group's perception of their physical and mental health over time [45], is of major importance in people with HHT [3, 14, 46, 47] and this should be reflected in clinical trials. Previous studies have demonstrated that the symptoms with the greatest impact on the HRQoL of people with HHT are recurrent epistaxis and the consequences of chronic iron deficiency and iron deficiency anemia, namely fatigue [48]. The science of HRQoL measurement in HHT clinical trials remains in its early stages, however.

Generic HRQoL measurement scales, such as the Short Form‐36 (SF‐36) and Euro Quality of Life 5 Dimension scale (EuroQOL‐5D) have previously demonstrated reduced HRQoL in people with HHT relative to the general population, most notably with regards to anxiety, depression, pain, and discomfort [49, 50]. However, the generic scales included in major HHT clinical trials, such as PROMIS inventories and the SF‐12 have generally performed poorly, with limited or no correlation to epistaxis measures or HHT‐specific HRQoL measurements [19, 22]. This is not unexpected given that these generic scales have no epistaxis‐specific items. The HHT‐specific HRQoL measurement scales, Quality of Life Questionnaire in HHT and HHT‐Specific Quality of Life Scale [46, 47] (Appendix, pp.26–28) remain quite new. The Quality of Life Questionnaire in HHT has been validated, but not yet utilized in a clinical trial; the HHT‐Specific Quality of Life Scale recently underwent validation in an interventional clinical trial (PATH‐HHT), and the formal manuscript describing this validation is awaited. As is true for many diseases, given the differences between how children and adults experience disease, the expert panel acknowledges the need for a pediatric HHT‐specific HRQoL measurement scale to be developed and validated.

Therefore, at this time, the expert panel recommends the inclusion of HHT‐specific HRQoL measurement scales in all prospective interventional HHT clinical trials, with the caveat that until validated in an interventional clinical trial such a measurement should be designated a secondary or exploratory endpoint. Inclusion of generic HRQoL measurement scales may be considered in addition to the use of HHT‐specific scales but should be designated non‐key secondary or exploratory endpoints.

## 8 Bleeding Response Criteria and Clinically Important Differences in Clinical Studies

At present, beyond the minimal clinically important difference (MCID) for the ESS and NOSE HHT scores, there remains considerable heterogeneity in what clinical studies determine to be a clinically important difference in epistaxis and hematologic endpoints. Defining epistaxis and hematologic response thresholds as well as defining a clinically meaningful improvement between groups within a randomized study based on the best available evidence and the expertise of the panel was therefore considered an essential component of this standardization process. Importantly, all criteria defined in this section are intended for use in clinical studies and should not be used by payors to define response or lack thereof to a therapy in clinical practice.

Epistaxis endpoints can be statistically assessed in multiple ways. Repeated measures models can compare improvements in study groups over time, as was done with the ESS in the PATH‐HHT trial and with epistaxis diary measurements in the VAD044002 trial [19, 24]. Conversely, although disadvantageous from a statistical power perspective, individual participants may be classified in binary fashion as achieving an epistaxis response or as non‐responders, and groups analyzed on the basis of responder proportions, as was done in the EPICURE trial [22].

### 8.1 Epistaxis Response Definitions

The expert panel defined the terms epistaxis response (ER), optimal epistaxis response (OER), clinical epistaxis improvement (CEI), nonresponse (NR), and durable response (durable ER and durable OER), Table 3, for responder analysis (response to treatment) of epistaxis diary data (frequency, duration, and severity). Loss of response and loss of optimal response were additionally defined. The expert panel determined that these definitions should be generally applicable across HHT‐associated bleeding clinical studies and, if binary responder analysis is chosen as an efficacy endpoint, recommends their use (versus alternative study‐specific response definitions) unless there is a uniquely compelling reason not to do so on the basis of the individual drug/intervention and its toxicities. Additionally, the risk–benefit profile of a specific therapy should be considered when determining the response definition used as the primary response threshold. For most therapeutics, expected to have mild to moderate toxicity, ER may be appropriate as the threshold for response, and for those with more intensive possible toxicities and/or cost (such as a potential cell or gene therapeutic), OER may be more appropriate as the primary response threshold. Illustrative examples of proper application of these response definitions are given in the Appendix, p.29.

**TABLE 3:** Epistaxis response definitions for responder analysis in individual participants.

Term	Definition	Interval required to adjudicate	Notes
Epistaxis Response (ER)	Reduction in epistaxis duration, frequency, or a composite epistaxis endpoint (e.g., intensity‐adjusted duration) of 30%–50% relative to baseline	At least 1 month (28 days) worth of epistaxis diary data required in order to adjudicate	*Loss of response* is defined as dropping below the 30% improvement (relative to baseline) threshold in the epistaxis parameter for at least 2 consecutive months (56 days)
Optimal Epistaxis Response (OER)	Reduction in epistaxis duration, frequency, or a composite epistaxis endpoint (e.g., intensity‐adjusted duration) of > 50% relative to baseline	At least 1 month (28 days) worth of epistaxis diary data required in order to adjudicate	*Loss of optimal response* is defined as dropping below the > 50% improvement (relative to baseline) threshold in the epistaxis parameter for at least 2 consecutive months (56 days)
Nonresponse (NR)	Failure to achieve ER	At least 1 month (28 days) worth of epistaxis diary data required in order to adjudicate	A participant classified as NR may be given the sub‐designation of *Clinical Epistaxis Improvement (CEI)*, defined as achieving an objective epistaxis benefit despite not achieving criteria for ER (e.g., measurable improvement in epistaxis intensity)
Durable Response	Achievement of ER (without loss of response) or OER (without loss of optimal response) for a period of 6 months or longer	At least 6 months (168 days) worth of epistaxis diary data required in order to adjudicate	Intended for use primarily in open‐label extension studies; can be defined as durable ER or durable OER

*Note*: The definitions listed in this table may be used in relation to any prespecified epistaxis diary measurement, including epistaxis duration, frequency, or a composite epistaxis endpoint (e.g., intensity‐adjusted duration). Currently, the most optimal epistaxis diary parameter(s) (e.g., frequency versus duration versus a composite) for application to binary responder analysis are not known and are a topic of ongoing investigation.

### 8.2 Clinically Important Difference for Epistaxis Parameters in Randomized Trials

After an in‐depth review of the randomized clinical trials completed over the 20 years preceding this report (Appendix, pp.7–9), the expert panel defines a minimum difference in an epistaxis diary endpoint (frequency, duration, or a composite endpoint such as intensity‐adjusted duration) of 15% or more between the mean outcome scores of two groups (an experimental group and a placebo group, or two treatment groups undergoing comparison) as a clinically meaningful difference between groups. This number is consistent with the magnitude of benefit observed between arms in successful large placebo‐controlled studies that have been done of efficacious therapeutics such as systemic tranexamic acid and pomalidomide [19, 25], considers that a significant proportion of participants with this heterogeneous disease may be non‐responders to an otherwise efficacious therapeutic and accounts for the well‐documented placebo effect with epistaxis measurements. Additionally, the expert panel recognizes a minimum difference between groups in a validated epistaxis severity instrument (e.g., ESS or NOSE HHT) to be equal to the MCID of the instrument (0.71 points in the case of the ESS, and 0.46 points in the case of the NOSE HHT) [40, 51].

### 8.3 Addressing Baseline Epistaxis Outliers

Outliers with regards to baseline monthly epistaxis duration or frequency are common in HHT clinical trials (Appendix, pp.10–13), which increase variability in measurements, skew pre‐ and post‐treatment means, and impose additional challenges in statistical planning and analysis [52]. The expert panel recommends including, in the prespecified statistical analysis plan for all clinical trials, a standard statistical approach to addressing outliers (on the basis of baseline epistaxis measurements) in primary efficacy analyses, or at least including a prespecified sensitivity analysis in which these outliers are addressed. The approach should be selected based on what is most appropriate and rigorous for the specific study in consultation with a biostatistician.

### 8.4 Hematologic Response Definitions

The expert panel defined the terms hematologic response (HR), optimal hematologic response (OHR), hemoglobin response (HbR), and optimal hemoglobin response (OHbR), Table 4. Hematologic support dependence/freedom and red cell transfusion dependence/freedom are additionally defined.

**TABLE 4:** Hematologic response definitions and other hematologic definitions.

Term	Definition	Interval required to adjudicate	Notes
Hematologic Response (HR)	Improvement in hematologic support requirements (intravenous iron, red cell transfusion) of 30%–50% relative to baseline	At least 12 weeks (84 days) worth of data pre‐ and post‐treatment required in order to adjudicate	May evaluate composite hematologic endpoints (e.g., HSS, measured in RUEs) or solitary hematologic endpoints (e.g., milligrams of iron infused, units red cells transfused) Must compare a baseline/pretreatment interval of at least 12 weeks with a posttreatment interval of at least 12 weeks
Optimal Hematologic Response (OHR)	Improvement in hematologic support requirements (intravenous iron, red cell transfusion) of > 50% relative to baseline	At least 12 weeks (84 days) worth of data pre‐ and post‐treatment required in order to adjudicate	May evaluate composite hematologic endpoints (e.g., HSS, measured in RUEs) or solitary hematologic endpoints (e.g., milligrams of iron infused, units red cells transfused) Must compare a baseline/pretreatment interval of at least 12 weeks with a posttreatment interval of at least 12 weeks
Hemoglobin Response (HbR)^a^	Improvement in hemoglobin of ≥ 1.0 g/dL while all other hematologic support remains stable or decreases	At least 12 weeks (84 days) worth of data required in order to adjudicate	Must compare a baseline/pretreatment hemoglobin value(s) with a posttreatment hemoglobin value(s) over a treatment interval of at least 12 weeks During the evaluated interval, oral iron, intravenous iron, and red cell transfusion administration must remain stable or decrease while the posttreatment hemoglobin improves
Optimal Hemoglobin Response (OHbR)^a^	Improvement in hemoglobin of ≥ 2.0 g/dL while all other hematologic support remains stable or decreases	At least 12 weeks (84 days) worth of data required in order to adjudicate	Must compare a baseline/pretreatment hemoglobin value(s) with a posttreatment hemoglobin value(s) over a treatment interval of at least 12 weeks During the evaluated interval, oral iron, intravenous iron, and red cell transfusion administration must remain stable or decrease while the posttreatment hemoglobin improves
Other Hematologic Definitions			
Hematologic Support Dependence	Requirement for an average of ≥ 1 RUE per month in hematologic support to maintain a normal hemoglobin (at least 12 weeks [84 days] worth of data required in order to adjudicate). An inability to maintain a normal hemoglobin despite receiving an average of ≥ 1 RUE per month, while not meeting criteria for red cell transfusion dependence (see below), also qualifies as hematologic support dependence.		
Hematologic Support Free	No intravenous iron or red cell transfusions given for ≥ 6 months while maintaining a normal hemoglobin		
Red Cell Transfusion Dependence	Requirement for an average of ≥ 2 units of red cells transfused per month to maintain an acceptable (i.e., safe) hemoglobin (at least 12 weeks [84 days] worth of data required in order to adjudicate)		
Red Cell Transfusion Free	No red cells transfused for ≥ 6 months while maintaining an acceptable hemoglobin		

Abbreviations: HIS, Hematologic Impact Score; HSS, Hematologic Support Score; RUEs, red‐cell unit equivalents.

^a^ Hemoglobin responses HbR and OHbR should only be evaluated in participants who are sufficiently anemic at baseline (e.g., hemoglobin < 11 g/dL).

### 8.5 Health‐Related Quality of Life Response Definitions

As outlined in Section 7, the expert panel recognizes the importance of HRQoL measurements in HHT clinical trials. However, as discussed in Section 7, the science of HRQoL measurement in HHT clinical trials remains in its early stages. Therefore, the expert panel determined this as an important area of future research and not currently appropriate for formalized response definitions.

### 8.6 Treatment Refractoriness

The expert panel defined “refractoriness” in the context of HHT‐associated bleeding to apply to individual treatments only. The expert panel highlighted that it should be used to describe the symptom or disease state (not the person), and that it should be used in the context of advanced systemic therapies (e.g., systemic antiangiogenic agents, such as bevacizumab, thalidomide, pomalidomide, and pazopanib) only. For example, a person's HHT may be refractory to bevacizumab and pazopanib but responsive to pomalidomide. A person's HHT is refractory to a certain therapeutic if they no longer derive clinical benefit from that agent or have never derived clinical benefit from that agent. Clinical benefit in this circumstance is best defined by the participant and treating investigator. This terminology is best suited to use in eligibility determination for clinical trials, e.g., a trial seeking to enroll participants with bleeding refractory to bevacizumab.

## 9 Conclusions

This ICR harmonizes definitions, terminology, and response criteria for bleeding in HHT, providing an evidence‐ and expertise‐based framework for the development and implementation of clinical studies in this field. It is also expected to be useful in the future development of clinical guidelines. This was achieved through the use of rigorous evidence review, a large and international group of experts, inclusion of the patient and patient advocate voice, achievement of consensus over multiple face‐to‐face meetings and multiple rounds of review and revision, and rigorous external review prior to finalization. It stands out as the largest effort to date in the global harmonization of HHT terminology, definitions, and clinical trial planning and design. Ultimately, standardization efforts such as this one must evolve with time and acquisition of new evidence, and the ICR Working Group members are committed to the ongoing re‐evaluation of this report.

## Author Contributions

H.Al‐Samkari contributed to conception and design, data collection, analysis and interpretation of the data, writing the first draft of the manuscript, critical revision of the intellectual content, final approval of the manuscript, and administrative, technical, and logistic support. M. E. Faughnan, R. S. Kasthuri, H. J. Mager, J. Y. Zhou, M. M. Serra, B. T. Samuelson‐Bannow, L. N. Van Doren, J. F. Piccirillo, and M. S. Clancy contributed to data collection, analysis and interpretation of the data, critical revision of the intellectual content, and final approval of the manuscript. All other authors contributed to analysis and interpretation of the data, critical revision of the intellectual content, and final approval of the manuscript.

## Ethics Statement

The authors have nothing to report.

## Conflicts of Interest

No author received any financial support or compensation from any source for this report/manuscript. H. Al‐Samkari: Grants or contracts (research funding to institution) (Agios, Sobi, Vaderis, Novartis, Amgen), consulting fees (Agios, Amgen, Alnylam, Alpine, Sobi, argenx, Pharmacosmos, Novartis, Sanofi). A. Hammill: Grants or contracts (research funding to institution) (Venthera, Merck, Novartis, Protara), consulting fees (Novartis, Relay, Diagonal, Ipsen, Aytu, Ideaya), support for meetings/travel (Cure HHT, Relay), patents planned, issued or pending (Application #18/746610: “Sirolimus pharmacokinetics guided and model informed precision dosing”, filed 06/18/2024), participation in data safety monitoring board/advisory board (NHLBI DSMB for PATH‐HHT trial, 2019–2023), leadership in other board/society/committee, paid or unpaid (ASPHO Vascular Anomalies Special Interest Group Chair 2022–2024, Vice Chair 2020–2022, unpaid). S. Olitsky: Consulting fees (Pharmacosmos). M. Clancy: Consulting fees (Alnylam). H. J. Mager: Grants or contracts (Vaderis), payment or honoraria for lectures/presentations/other (grant paid to Kees Westermann Foundation, not to Dr. Mager specifically). R. Kasthuri: Grants or contracts (research funding to institution) (NIH, DOD, HRSA), consulting fees (Alnylam, Crosswalk, Pharmacosmos, Tectonic). M. Faugnan: Grants or contracts (research funding to institution) (Vaderis, NIH, DOD), consulting fees (Biomarin, Alphasights, Ipsen, Alnylam, Tectonic), participation in data safety monitoring board/advisory board (NHLBI DSMB for PATH‐HHT trial, 2019–2023). B. Samuelson‐Bannow: Consulting fees (Hemagiologics). L. Van Doren: Consulting fees (Sanofi, Sobi, Pharmacosmos), payment or honoraria for lectures/presentations/other (Pharmacosmos, Daiichi Sankyo, Sanofi, Global Blood Therapeutics/Pfizer, Sobi), payment for expert testimony (Massachusetts Medical Board), support for meetings/travel (American Society of Hematology), leadership in other board/society/committee (American Society of Hematology Guidelines on Iron Deficiency, Fem Foundation). J. Piccirillo: Royalties or licenses (NOSE HHT, receives royalty payments from his employer, Washington University, when they license use of NOSE HHT to commercial interests). J. Zhou: Consulting fees (Takeda, Pharmacosmos, Diagonal). S. Thomas: Grants or contracts (research funding to institution) (NHLBI). K. McCrae: Payment or honoraria for lectures/presentations/other (MD Anderson Cancer Center, University of Southern California), participation in data safety monitoring board/advisory board (Alnylam). S. Sewaralthahab: Consulting fees (Bristol‐Myers Squibb, Pfizer), payment or honoraria for lectures/presentations/other (Bristol‐Myers Squibb, Pfizer, Novo Nordisk), participation in data safety monitoring board/advisory board (Bristol‐Myers Squibb). A. Von Drygalski: Consulting fees (Biomarin, Regeneron, Pfizer, Sobi, Sanofi, CSL Behring, Novo Nordisk, Sparkx, Takeda, Genentech), leadership in other board/society/committee, paid or unpaid (Hematherix). A. Pishko: Grants or contracts (research funding to institution) (ANTHOS, Janssen, NIH/NHLBI), royalties or licenses (UpToDate), participation in data safety monitoring board/advisory board (Biomarin). J. Gossage: Consulting fees (Vaderis), participation in data safety monitoring board/advisory board (DSMB chair, Tacrolimus in HHT bleeding trial and Doxycycline in HHT bleeding trial, both U.S. federally funded trials). All other authors report no conflicts of interest. The authors certify that they do not have, within the past 3 years or with a relevant company or competitor, any stocks or shares, equity, a contract of employment, or a named position on a company board; hold (or are applying for) a relevant patent; or have been asked by anyone to write, be named on, or to submit this manuscript.

## Supporting information


**Data S1.** Supplementary Appendix.

## Data Availability

The data that support the findings of this study are available from the corresponding author upon reasonable request.

## References

[1] A. D. Kjeldsen , P. Vase , and A. Green , “Hereditary Haemorrhagic Telangiectasia: A Population‐Based Study of Prevalence and Mortality in Danish Patients,” Journal of Internal Medicine 245, no. 1 (1999): 31–39, 10.1046/j.1365-2796.1999.00398.x. 10095814

[2] E. Zhang , Z. M. Virk , J. Rodriguez‐Lopez , and H. Al‐Samkari , “Hereditary Hemorrhagic Telangiectasia May be the Most Morbid Inherited Bleeding Disorder in Women,” Blood Advances 8, no. 12 (2024): 3166–3172, 10.1182/bloodadvances.2023011961. 38593443 PMC11225659

[3] S. Chaturvedi , M. Clancy , N. Schaefer , O. Oluwole , and K. R. McCrae , “Depression and Post‐Traumatic Stress Disorder in Individuals With Hereditary Hemorrhagic Telangiectasia: A Cross‐Sectional Survey,” Thrombosis Research 153 (2017): 14–18, 10.1016/j.thromres.2017.03.003. 28314138 PMC5420428

[4] O. S. AAssar , C. M. Friedman , and R. I. White, Jr. , “The Natural History of Epistaxis in Hereditary Hemorrhagic Telangiectasia,” Laryngoscope 101, no. 9 (1991): 977–980, 10.1288/00005537-199109000-00008. 1886446

[5] A. D. Kjeldsen and J. Kjeldsen , “Gastrointestinal Bleeding in Patients With Hereditary Hemorrhagic Telangiectasia,” American Journal of Gastroenterology 95, no. 2 (2000): 415–418, 10.1111/j.1572-0241.2000.01792.x. 10685743

[6] R. Ola , J. Hessels , A. Hammill , et al., “Executive Summary of the 14th HHT International Scientific Conference,” Angiogenesis 26, no. Suppl 1 (2023): 27–37, 10.1007/s10456-023-09886-5. 37695357 PMC10543799

[7] HHT , “2022 14th HHT International Scientific Conference Abstracts,” Angiogenesis 26, no. Suppl 1 (2023): 1–25, 10.1007/s10456-023-09887-4. 37682429

[8] R. S. Kasthuri , M. Montifar , J. Nelson , et al., “Prevalence and Predictors of Anemia in Hereditary Hemorrhagic Telangiectasia,” American Journal of Hematology 92, no. 10 (2017): E591–E593, 10.1002/ajh.24832. PMC5997494 28639385

[9] H. Al‐Samkari , T. J. Mayne , M. Troutt , H. Patle , M. Clancy , and E. Duhaime , “Characterizing the Healthcare Utilization and Costs of Hereditary Hemorrhagic Telangiectasia,” American Journal of Hematology 100, no. 10 (2025): 1722–1735, 10.1002/ajh.27756. PMC12417757 40600700

[10] A. Kritharis , H. Al‐Samkari , and D. J. Kuter , “Hereditary Hemorrhagic Telangiectasia: Diagnosis and Management From the Hematologist's Perspective,” Haematologica 103, no. 9 (2018): 1433–1443, 10.3324/haematol.2018.193003. 29794143 PMC6119150

[11] C. L. Shovlin , E. Buscarini , C. Sabba , et al., “The European Rare Disease Network for HHT Frameworks for Management of Hereditary Haemorrhagic Telangiectasia in General and Speciality Care,” European Journal of Medical Genetics 65, no. 1 (2022): 104370, 10.1016/j.ejmg.2021.104370. 34737116

[12] J. W. Donaldson , T. M. McKeever , I. P. Hall , R. B. Hubbard , and A. W. Fogarty , “Complications and Mortality in Hereditary Hemorrhagic Telangiectasia: A Population‐Based Study,” Neurology 84, no. 18 (2015): 1886–1893, 10.1212/WNL.0000000000001538. 25862798 PMC4433463

[13] K. P. Thompson , J. Nelson , H. Kim , et al., “Predictors of Mortality in Patients With Hereditary Hemorrhagic Telangiectasia,” Orphanet Journal of Rare Diseases 16, no. 1 (2021): 12, 10.1186/s13023-020-01579-2. 33407668 PMC7789194

[14] M. E. Faughnan , J. J. Mager , S. W. Hetts , et al., “Second International Guidelines for the Diagnosis and Management of Hereditary Hemorrhagic Telangiectasia,” Annals of Internal Medicine 173, no. 12 (2020): 989–1001, 10.7326/M20-1443. 32894695

[15] M. E. Faughnan , J. J. Mager , S. W. Hetts , V. A. Palda , and F. Ratjen , “Second International Guidelines for the Diagnosis and Management of Hereditary Hemorrhagic Telangiectasia,” Annals of Internal Medicine 174, no. 7 (2021): 1035–1036, 10.7326/L21-0067. 34280351

[16] H. Al‐Samkari , “Hereditary Hemorrhagic Telangiectasia: Systemic Therapies, Guidelines, and an Evolving Standard of Care,” Blood 137, no. 7 (2021): 888–895, 10.1182/blood.2020008739. 33171488

[17] H. Al‐Samkari , R. S. Kasthuri , J. G. Parambil , et al., “An International, Multicenter Study of Intravenous Bevacizumab for Bleeding in Hereditary Hemorrhagic Telangiectasia: The InHIBIT‐Bleed Study,” Haematologica 106, no. 8 (2021): 2161–2169, 10.3324/haematol.2020.261859. 32675221 PMC8327711

[18] R. Invernizzi , F. Quaglia , C. Klersy , et al., “Efficacy and Safety of Thalidomide for the Treatment of Severe Recurrent Epistaxis in Hereditary Haemorrhagic Telangiectasia: Results of a Non‐Randomised, Single‐Centre, Phase 2 Study,” Lancet Haematology 2, no. 11 (2015): e465–e473, 10.1016/S2352-3026(15)00195-7. 26686256 PMC4839500

[19] H. Al‐Samkari , R. S. Kasthuri , V. N. Iyer , et al., “Pomalidomide for Epistaxis in Hereditary Hemorrhagic Telangiectasia,” New England Journal of Medicine 391, no. 11 (2024): 1015–1027, 10.1056/NEJMoa2312749. 39292928 PMC11412318

[20] J. G. Parambil , J. R. Gossage , K. R. McCrae , et al., “Pazopanib for Severe Bleeding and Transfusion‐Dependent Anemia in Hereditary Hemorrhagic Telangiectasia,” Angiogenesis 25, no. 1 (2022): 87–97, 10.1007/s10456-021-09807-4. 34292451 PMC8295629

[21] R. Torres‐Iglesias , J. M. Mora‐Lujan , A. Iriarte , P. Cerdà , E. Alba , and M. Á. Sánchez‐Corral , “Long‐Term Use of Somatostatin Analogs for Chronic Gastrointestinal Bleeding in Hereditary Hemorrhagic Telangiectasia,” Frontiers in Medicine (Lausanne) 10 (2023): 1146080, 10.3389/fmed.2023.1146080. PMC10213420 37250655

[22] R. Hermann , V. Grobost , X. Le‐Guillou , et al., “Effect of Oral Nintedanib vs Placebo on Epistaxis in Hereditary Hemorrhagic Telangiectasia: The Epicure Multicenter Randomized Double‐Blind Trial,” Angiogenesis 28, no. 1 (2024): 9, 10.1007/s10456-024-09962-4. 39718659 PMC11668894

[23] S. Dupuis‐Girod , S. Riviere , C. Lavigne , et al., “Efficacy and Safety of Intravenous Bevacizumab on Severe Bleeding Associated With Hemorrhagic Hereditary Telangiectasia: A National, Randomized Multicenter Trial,” Journal of Internal Medicine 294, no. 6 (2023): 761–774, 10.1111/joim.13714. 37592715

[24] H. Al‐Samkari , J. Hessels , A. Riera‐Mestre , et al., “A Randomized, Placebo‐Controlled, Multicenter Proof‐of‐Concept (POC) Study to Assess the Safety and Efficacy of the Novel Allosteric AKT Inhibitor, VAD044, in Adults With Hereditary Hemorrhagic Telangiectasia (HHT),” Blood 144, no. Suppl 1 (2024): 553.

[25] S. Gaillard , S. Dupuis‐Girod , F. Boutitie , et al., “Tranexamic Acid for Epistaxis in Hereditary Hemorrhagic Telangiectasia Patients: A European Cross‐Over Controlled Trial in a Rare Disease,” Journal of Thrombosis and Haemostasis 12, no. 9 (2014): 1494–1502, 10.1111/jth.12654. 25040799

[26] U. W. Geisthoff , U. T. Seyfert , M. Kubler , B. Bieg , P. K. Plinkert , and J. Konig , “Treatment of Epistaxis in Hereditary Hemorrhagic Telangiectasia With Tranexamic Acid—A Double‐Blind Placebo‐Controlled Cross‐Over Phase IIIB Study,” Thrombosis Research 134, no. 3 (2014): 565–571, 10.1016/j.thromres.2014.06.012. 25005464

[27] H. Al‐Samkari , “Giving Hereditary Haemorrhagic Telangiectasia the Attention it Deserves,” Lancet Haematology 8, no. 7 (2021): e472–e474, 10.1016/S2352-3026(21)00164-2. 34171274

[28] S. Dupuis‐Girod , I. Ginon , J. C. Saurin , et al., “Bevacizumab in Patients With Hereditary Hemorrhagic Telangiectasia and Severe Hepatic Vascular Malformations and High Cardiac Output,” JAMA 307, no. 9 (2012): 948–955, 10.1001/jama.2012.250. 22396517

[29] H. Al‐Samkari , H. A. Albitar , S. E. Olitsky , M. S. Clancy , and V. N. Iyer , “Systemic Bevacizumab for High‐Output Cardiac Failure in Hereditary Hemorrhagic Telangiectasia: An International Survey of HHT Centers,” Orphanet Journal of Rare Diseases 14, no. 1 (2019): 256, 10.1186/s13023-019-1239-6. 31727111 PMC6857247

[30] J. McDonald , P. Bayrak‐Toydemir , and R. E. Pyeritz , “Hereditary Hemorrhagic Telangiectasia: An Overview of Diagnosis, Management, and Pathogenesis,” Genetics in Medicine 13, no. 7 (2011): 607–616, 10.1097/GIM.0b013e3182136d32. 21546842

[31] L. Van Doren , M. Steinheiser , K. Boykin , K. J. Taylor , M. Menendez , and M. Auerbach , “Expert Consensus Guidelines: Intravenous Iron Uses, Formulations, Administration, and Management of Reactions,” American Journal of Hematology 99, no. 7 (2024): 1338–1348, 10.1002/ajh.27220. 38282557

[32] J. B. Hoag , P. Terry , S. Mitchell , D. Reh , and C. A. Merlo , “An Epistaxis Severity Score for Hereditary Hemorrhagic Telangiectasia,” Laryngoscope 120, no. 4 (2010): 838–843, 10.1002/lary.20818. 20087969

[33] A. M. Peterson , D. Kallogjeri , E. Spitznagel , M. M. Chakinala , J. S. Schneider , and J. F. Piccirillo , “Development and Validation of the Nasal Outcome Score for Epistaxis in Hereditary Hemorrhagic Telangiectasia (NOSE HHT),” JAMA Otolaryngology. Head & Neck Surgery 146, no. 11 (2020): 999–1005, 10.1001/jamaoto.2020.3040. 33022056 PMC7530813

[34] K. J. Whitehead , N. B. Sautter , J. P. McWilliams , et al., “Effect of Topical Intranasal Therapy on Epistaxis Frequency in Patients With Hereditary Hemorrhagic Telangiectasia: A Randomized Clinical Trial,” JAMA 316, no. 9 (2016): 943–951, 10.1001/jama.2016.11724. 27599329

[35] H. Al‐Samkari , “How I Treat Bleeding in Hereditary Hemorrhagic Telangiectasia,” Blood 144, no. 9 (2024): 940–954, 10.1182/blood.2023021765. 38864625 PMC11830975

[36] H. Al‐Samkari , R. P. Naik , and N. A. Zakai , “A Hematologic Support Score for Longitudinal Measurement of Blood and Iron Requirements in Hereditary Hemorrhagic Telangiectasia and Other Chronic Bleeding Disorders,” Research and Practice in Thrombosis and Haemostasis 4, no. 8 (2020): 1340–1342, 10.1002/rth2.12444. 33313473 PMC7695564

[37] S. Schulman and C. Kearon , “Subcommittee on Control of Anticoagulation of the S, Standardization Committee of the International Society on T, Haemostasis. Definition of Major Bleeding in Clinical Investigations of Antihemostatic Medicinal Products in Non‐Surgical Patients,” Journal of Thrombosis and Haemostasis 3, no. 4 (2005): 692–694, 10.1111/j.1538-7836.2005.01204.x. 15842354

[38] A. M. Peterson , M. M. Chakinala , and J. F. Piccirillo , “A Framework for Clinical Trials in Hereditary Hemorrhagic Telangiectasia‐Associated Epistaxis‐Navigating the PATH,” JAMA Otolaryngology. Head & Neck Surgery 151 (2025): 425–426, 10.1001/jamaoto.2025.0017. 40079979

[39] P. Edwards , “Questionnaires in Clinical Trials: Guidelines for Optimal Design and Administration,” Trials 11 (2010): 2, 10.1186/1745-6215-11-2. 20064225 PMC2823735

[40] A. M. Peterson , J. J. Lee , D. Kallogjeri , J. S. Schneider , M. M. Chakinala , and J. F. Piccirillo , “Efficacy of Timolol in a Novel Intranasal Thermosensitive Gel for Hereditary Hemorrhagic Telangiectasia‐Associated Epistaxis: A Randomized Clinical Trial,” JAMA Otolaryngology. Head & Neck Surgery 146, no. 11 (2020): 1006–1014, 10.1001/jamaoto.2020.3025. 32940653 PMC7499238

[41] H. Al‐Samkari , “Even Effective Drugs Require Adequately Powered Trials: Systemic Bevacizumab in Hereditary Hemorrhagic Telangiectasia,” Journal of Internal Medicine 294, no. 6 (2023): 684–686, 10.1111/joim.13713. 37608622 PMC10841238

[42] A. Glenthoj , E. J. van Beers , H. Al‐Samkari , V. Viprakasit , K. H. M. Kuo , and F. Galactéros , “Mitapivat in Adult Patients With Pyruvate Kinase Deficiency Receiving Regular Transfusions (Activate‐T): A Multicentre, Open‐Label, Single‐Arm, Phase 3 Trial,” Lancet Haematology 9, no. 10 (2022): e724–e732, 10.1016/S2352-3026(22)00214-9. 35988546

[43] J. M. Higham , P. M. O'Brien , and R. W. Shaw , “Assessment of Menstrual Blood Loss Using a Pictorial Chart,” British Journal of Obstetrics and Gynaecology 97, no. 8 (1990): 734–739, 10.1111/j.1471-0528.1990.tb16249.x. 2400752

[44] K. A. Matteson , D. M. Scott , C. A. Raker , and M. A. Clark , “The Menstrual Bleeding Questionnaire: Development and Validation of a Comprehensive Patient‐Reported Outcome Instrument for Heavy Menstrual Bleeding,” BJOG: An International Journal of Obstetrics & Gynaecology 122, no. 5 (2015): 681–689, 10.1111/1471-0528.13273. 25615842 PMC4373964

[45] WHO , “Study Protocol for the World Health Organization Project to Develop a Quality of Life Assessment Instrument (WHOQOL),” Quality of Life Research 2, no. 2 (1993): 153–159, https://www.ncbi.nlm.nih.gov/pubmed/8518769. 8518769

[46] T. T. T. Le , G. Martinent , S. Dupuis‐Girod , et al., “Development and Validation of a Quality of Life Measurement Scale Specific to Hereditary Hemorrhagic Telangiectasia: The QoL‐HHT,” Orphanet Journal of Rare Diseases 17, no. 1 (2022): 281, 10.1186/s13023-022-02426-2. 35854330 PMC9295423

[47] R. S. Kasthuri , S. Chaturvedi , S. Thomas , et al., “Development and Performance of a Hereditary Hemorrhagic Telangiectasia‐Specific Quality‐of‐Life Instrument,” Blood Advances 6, no. 14 (2022): 4301–4309, 10.1182/bloodadvances.2022007748. 35877137 PMC9327531

[48] G. Martinent , M. Carrot , A. Chirac , et al., “Hereditary Hemorrhagic Telangiectasia and Health‐Related Quality of Life: A Qualitative Investigation,” Quality of Life Research 29, no. 5 (2020): 1291–1299, 10.1007/s11136-020-02415-7. 31907872

[49] G. Pasculli , F. Resta , E. Guastamacchia , L. Di Gennaro , P. Suppressa , and C. Sabba , “Health‐Related Quality of Life in a Rare Disease: Hereditary Hemorrhagic Telangiectasia (HHT) or Rendu‐Osler‐Weber Disease,” Quality of Life Research 13, no. 10 (2004): 1715–1723, 10.1007/s11136-004-7865-y. 15651542

[50] U. W. Geisthoff , K. Heckmann , R. D'Amelio , et al., “Health‐Related Quality of Life in Hereditary Hemorrhagic Telangiectasia,” Otolaryngology–Head and Neck Surgery 136, no. 5 (2007): 726–733; Discussion 734–5, 10.1016/j.otohns.2006.12.019. 17478205

[51] L. X. Yin , D. D. Reh , J. B. Hoag , et al., “The Minimal Important Difference of the Epistaxis Severity Score in Hereditary Hemorrhagic Telangiectasia,” Laryngoscope 126, no. 5 (2016): 1029–1032, 10.1002/lary.25669. 26393959

[52] S. K. Kwak and J. H. Kim , “Statistical Data Preparation: Management of Missing Values and Outliers,” Korean Journal of Anesthesiology 70, no. 4 (2017): 407–411, 10.4097/kjae.2017.70.4.407. 28794835 PMC5548942

