# Supplementary material for: Standardization of Terminology, Definitions, and Outcome Criteria for Bleeding in Hereditary Hemorrhagic Telangiectasia: International Consensus Report
Source: Am J Hematol. 2025 Jul 15;100(10):1813–27. doi: 10.1002/ajh.70011 (PMC12417758; doi:10.1002/ajh.70011)
Supplement: Supplementary file 1 — Data S1. Supplementary Appendix. [file AJH-100-1813-s001.docx]

**
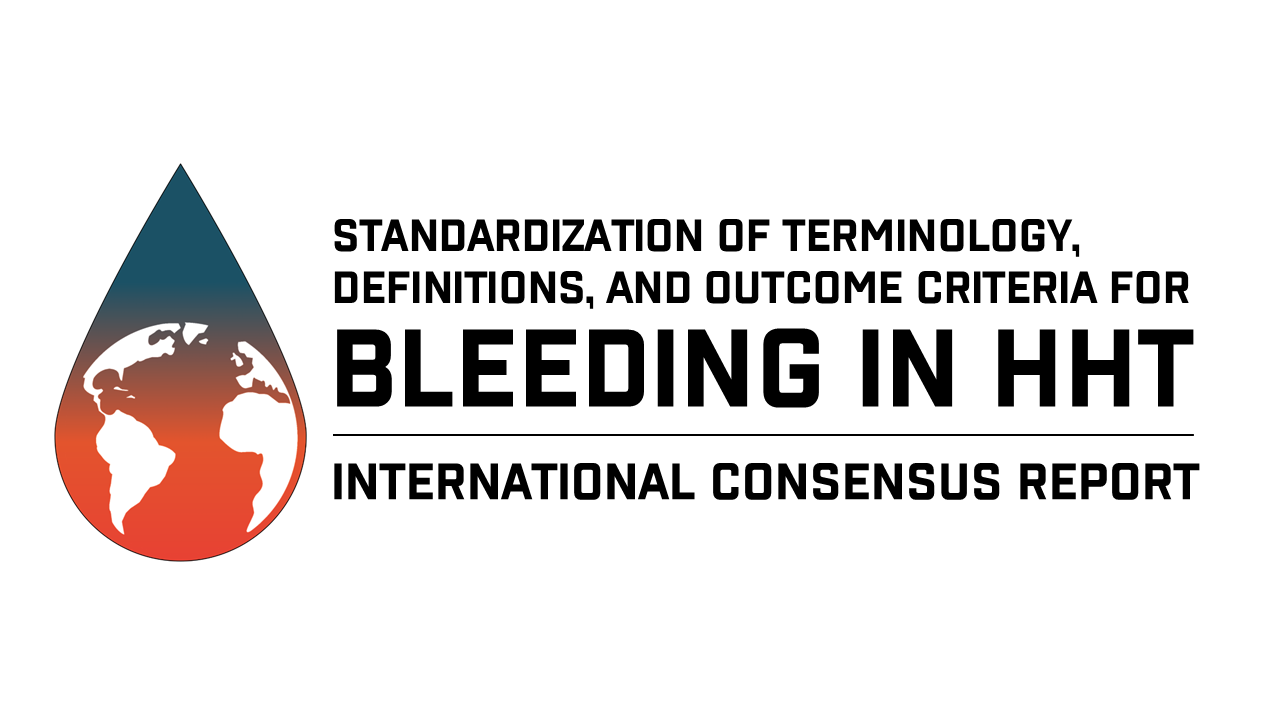
**

**STANDARDIZATION OF TERMINOLOGY, DEFINITIONS, AND OUTCOME CRITERIA FOR BLEEDING IN HEREDITARY HEMORRHAGIC TELANGIECTASIA: INTERNATIONAL CONSENSUS REPORT**

**SUPPLEMENTARY APPENDIX**

**TABLE OF CONTENTS**

International Consensus Report Working Group Members3

Systematic Literature Search and Identification of Evidence4

Evidence Tables7

Supplementary Table 17

Supplementary Table 210

External Review of Report: Process and Results14

**Details of Validated Epistaxis Severity Instruments Discussed in Report16**

Epistaxis Severity Score (ESS)16

Nasal Outcome Score for Epistaxis in Hereditary Hemorrhagic Telangiectasia (NOSE HHT)18

Justification for Numerical Thresholds in Bleeding Severity Classification22

Red-Cell Unit Equivalent (RUE) Background24

Pitfalls of Single/Solitary Hematologic Endpoints in HHT Trials25

**Details of HHT-Specific Health-Related Quality of Life Instruments Discussed in Report26**

HHT-Specific Quality of Life Scale (HHT-QoL) 26

Quality of Life Questionnaire in HHT (QoL-HHT) 27

**Illustrative Examples of Epistaxis Response Criteria29**

Supplementary Appendix References30

**INTERNATIONAL CONSENSUS REPORT WORKING GROUP MEMBERS**

| ***Name*** | ***Location, Country*** |
| --- | --- |
| Hanny Al-Samkari, M.D. (Chairperson, Steering Committee) | Massachusetts, USA |
| Raj Kasthuri, M.B.B.S. (Steering Committee) | North Carolina, USA |
| Hans-Jurgen Mager, M.D., Ph.D. (Steering Committee) | Niuewegein, Netherlands |
| Bethany Samuelson-Bannow, M.D., M.C.R. (Steering Committee) | Oregon, USA |
| Marie Faughnan, M.D. (Steering Committee) | Toronto, Canada |
| Marcelo Serra, M.D., Ph.D. (Steering Committee) | Buenos Aires, Argentina |
| Jay Piccirillo, M.D. (Steering Committee) | Missouri, USA |
| Layla Van Doren, M.D., M.B.A. (Steering Committee) | Connecticut, USA |
| Jenny Zhou, M.D. (Steering Committee) | California, USA |
| Marianne Clancy, M.P.A. (Patient/Foundation Representative, Steering Committee) | Maryland, USA |
| Keith McCrae, M.D. | Ohio, USA |
| Cedric Hermans, M.D., Ph.D. | Brussels, Belgium |
| Annette von Drygalski, M.D., PharmD | California, USA |
| Ingrid Winship, M.B.Ch.B., M.D. | Melbourne, Australia |
| Vivek Iyer, M.D., M.P.H. | Minnesota, USA |
| Allyson Pishko, M.D., M.S.C.E. | Pennsylvania, USA |
| Sarah Sewaralthahab, M.B.B.S., M.P.H. | Riyadh, Saudi Arabia |
| Sonia Thomas, Dr.P.H. (Biostatistician) | North Carolina, USA |
| Scott Olitsky, M.D., M.B.A. (Patient/Foundation Representative, Caregiver) | California, USA |
| Antoni Riera-Mestre, M.D., Ph.D. | Barcelona, Spain |
| Meir Mei-Zahav, M.D. | Tel Aviv, Israel |
| James Gossage, M.D. | Georgia, USA |
| Adrienne Hammill, M.D., Ph.D. | Ohio, USA |

**SYSTEMATIC LITERATURE SEARCH AND IDENTIFICATION OF EVIDENCE**

**Overview.** To support the development of evidence-based recommendations in this International Consensus Report, relevant literature was systematically identified, evaluated, and summarized into evidence tables for use by the ICR Working Group.

**Systematic Search and Identification of Evidence.** Two search strategies were developed and executed in Ovid MEDLINE on January 7, 2025, using a rubric developed by and under the guidance of medical librarians, to identify all relevant published studies in the past 20 years (2006-2025) relating to HHT-associated bleeding. Search strategies, which were designed for high sensitivity to ensure all relevant results were identified, combined both topic-specific controlled subject headings (MeSH terms) and relevant keywords in order to capture the broad topics of interest for each search (epistaxis and gastrointestinal bleeding). In total, 696 results were identified across both topics. H.A., the ICR Chairperson, reviewed the titles and abstracts, and based on the titles and abstracts 87 records that potentially met inclusion criteria were retrieved in full text for further review. Ultimately, the records meeting criteria for inclusion are summarized in the Evidence Tables below.

**Inclusion Criteria**:

- Patients diagnosed with hereditary hemorrhagic telangiectasia
- Intervention: Randomized trial of a therapeutic intervention to treat HHT-associated bleeding
- Comparison: Placebo or standard treatment
- English language publications
- Human subjects
- ≥ 5 subjects
- Original results published in an indexed journal (no data from published abstracts only included)

**Data Extraction and Appraisal.** Key data from the included studies was extracted and summarized in evidence tables to allow for inclusion of high quality published evidence in the report in a transparent manner.

**Search 1: Epistaxis**

| 1 | telangiectasia, hereditary hemorrhagic/ | 3723 |
| --- | --- | --- |
| 2 | hereditary h$emorrhagic telangiectasia$.mp. [mp=title, abstract, original title, name of substance word, subject heading word, floating sub-heading word, keyword heading word, organism supplementary concept word, protocol supplementary concept  word, rare disease supplementary concept word, unique identifier, synonyms] | 2577 |
| 3 | (Osler adj2 Weber adj2 Rendu).mp. [mp=title, abstract, original title, name of substance word, subject heading word, floating subheading word, keyword heading word, organism supplementary concept word, protocol supplementary concept word, rare disease  supplementary concept word, unique identifier, synonyms] | 1106 |
| 4 | or/1-3 | 4638 |
| 5 | limit 4 to (english language and humans and yr="2006- 2025") | 1811 |
| 6 | epistaxis/ | 5382 |
| 7 | (nose$ or nasal).mp. [mp=title, abstract, original title, name of substance word, subject heading word, floating sub-heading word, keyword heading word, organism supplementary concept word, protocol supplementary concept word, rare disease supplementary concept word, unique identifier, synonyms] | 215405 |
| 8 | 5 and (6 or 7) | 452 |

**Search 2: Gastrointestinal Bleeding**

| 1 | telangiectasia, hereditary hemorrhagic/ | 3723 |
| --- | --- | --- |
| 2 | hereditary h$emorrhagic telangiectasia$.mp. [mp=title, abstract, original title, name of substance word, subject heading word, floating sub-heading word, keyword heading word, organism supplementary concept word, protocol supplementary concept  word, rare disease supplementary concept word, unique identifier, synonyms] | 2577 |
| 3 | (Osler adj2 Weber adj2 Rendu).mp. [mp=title, abstract, original title, name of substance word, subject heading word, floating subheading word, keyword heading word, organism supplementary concept word, protocol supplementary concept word, rare disease  supplementary concept word, unique identifier, synonyms] | 1106 |
| 4 | or/1-3 | 4638 |
| 5 | limit 4 to (english language and humans and yr="2006- 2025") | 1811 |
| 6 | (gastr$ or GI).mp. [mp=title, book title, abstract, original title, name of substance word, subject heading word, floating sub-heading word, keyword heading word, organism supplementary concept word, protocol supplementary concept word, rare disease supplementary concept word, unique identifier, synonyms, population supplementary concept word, anatomy supplementary concept word] | 1004837 |
| 7 | 5 and 6 | 240 |
| 8 | exp colonoscopy/ or exp endoscopy, gastrointestinal/ | 105270 |
| 9 | fecal immunochemical test$.mp. | 1608 |
| 10 | (colon cancer screen$ or screen$ for colon cancer or colorectal cancer screen$ or screen$ for colorectal cancer or colonoscopic screen$).mp. [mp=title, book title, abstract, original title, name of substance word, subject heading word, floating sub-heading word, keyword heading word, organism supplementary concept word, protocol supplementary concept word, rare disease supplementary concept word, unique identifier, synonyms, population supplementary concept word, anatomy supplementary concept word] | 10796 |
| 11 | 5 and (8 or 9 or 10) | 34 |
| 12 | 7 or 11 | 244 |

**PRISMA Flow Diagram**


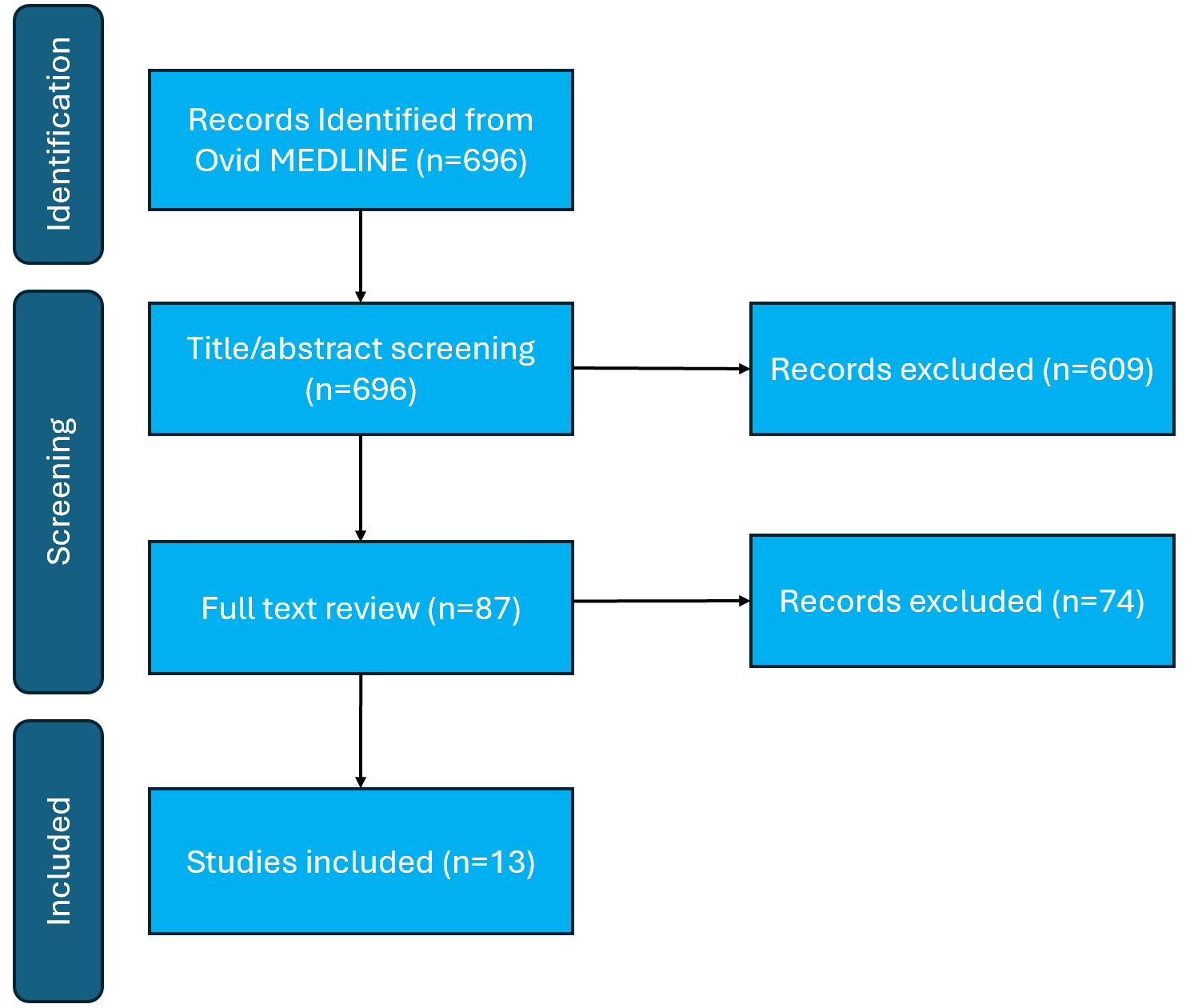


**EVIDENCE TABLES**

**Supplementary Table 1.** Summary of all HHT randomized trials for HHT-associated bleeding published between 2006 to 2025.

| **Study** | **Sample Size** | **Intervention** | **Design and Methods** | **Primary Outcome Measures** | **Primary Outcome Results** |
| --- | --- | --- | --- | --- | --- |
| Al-Samkari H. et al., *New Engl J Med* 2024^1^ | N=144 | Oral pomalidomide (4 mg per day) or matched placebo | RCT (double-blind, placebo-controlled). Treatment: 6 months, each | Epistaxis Severity Score change between pomalidomide and placebo, compared with baseline | Improved ESS in pomalidomide group relative to placebo exceeding the MCID (-0.94 difference, 95% confidence interval [CI], −1.57 to −0.31; *P*=0.004) |
| Boyer H. et al., *Int Forum Allergy Rhinol*  2015^2^ | N=17 | Sclerotherapy or control ("standard treatment", defined as continuation of any existing baseline treatment) | RCT (crossover) Treatment= 6 weeks, each  Washout period: None | Epistaxis Severity Score change in sclerotherapy period versus control period | Improved ESS in sclerotherapy period relative to control period (0.95 difference [SD 1.82], *P*=0.027). Treatment order was not statistically significant. |
| Dupuis-Girod S. et al., *JAMA* 2016^3^ | N=80 | Bevacizumab nasal spray (25 mg, 50 mg, or 75 mg) for 4 weeks or placebo nasal spray | RCT (double-blind, placebo-controlled) Treatment: 3 doses 14 days apart for a total treatment duration of 4 weeks | Mean monthly epistaxis duration for 3 months after end of treatment compared with 3 months before beginning of treatment | No statistical difference was observed in mean monthly epistaxis duration among treatment groups and placebo (*P*=.57) |
| Dupuis-Girod S. et al., *J Intern Med* 2023^4^ | N=23 | Intravenous bevacizumab (5 mg/kg) every 2 weeks for 3 months or placebo | RCT (double-blind, placebo-controlled)  Treatment: 6 doses each 2 weeks apart for a total treatment duration of 3 months | Proportion of red cell transfusion responders (participants achieving 50% reduction in units RBCs transfused during 3 months after treatment, compared with 3 months before treatment) | No statistically significant difference observed in proportion of responders in bevacizumab group (64%) versus placebo group (33%), *P*=0.22. |
| Gaillard S. et al., *J Thromb Haemost* 2014^5^ | N=135 | Oral tranexamic acid (3 g per day) or placebo | RCT (double-blind, placebo-controlled crossover):  Treatment: 3 months, each | Mean monthly epistaxis duration for the last 2 months of the treatment compared with the last 2 months of placebo | The mean duration of epistaxis per month was significantly shorter with tranexamic acid than placebo (0.19 on the log scale; SD=0.07; *P*=0.005). This difference corresponded to a decrease of 17.3% in the duration of epistaxis per month (95% CI, 5.5–27.6). |
| Geisthoff U.W. et al., *Thromb Res.* 2014^6^ | N=22 | Oral tranexamic acid (3g per day) or placebo | RCT (double-blind, placebo-controlled crossover): Treatment: 3 months, each. Washout period: None. | Change in hemoglobin (final minus initial) for each treatment period | No significant difference in change in hemoglobin between tranexamic acid and placebo (*P*=0.33, Mann–Whitney-U test).  Secondary endpoint of intensity-adjusted epistaxis duration was significantly improved (54% lower on TXA versus placebo) |
| Hermann R et al., *Angiogenesis* 2024^7^ | N=60 | Oral nintedanib (150 mg twice daily) or placebo | RCT (double-blind, placebo-controlled).  Treatment: 3 months, each | Proportion of epistaxis responders (participants achieving 50% reduction in epistaxis duration during the last 8 weeks of treatment) | Primary endpoint did not meet statistical significance (43% responders in nintedanib group versus 27% in placebo group, P=0.40)  Secondary endpoint of median epistaxis duration was significantly improved in nintedanib arm (57% reduction versus 24% in placebo arm, *P*=0.013) and decrease in median epistaxis frequency was also significant (3 vs. 12%, *P*=0.018) |
| McWilliams J. et al., *J Thromb Haemost* 2022^8^ | N=22 | Oral doxycycline hyclate 100 mg twice daily or placebo | RCT (double-blind, placebo-controlled crossover): Treatment: 2 months, each Washout period: 1 month | 3 co-primary endpoints: Epistaxis frequency, epistaxis duration, and ESS | None of the primary (or secondary) endpoints was statistically significantly improved  Most of the measured difference in epistaxis duration, and about half of the difference in epistaxis frequency, between the two groups came from worsening of the placebo group from baseline rather than improvement of the doxycycline group |
| Mei-Zahav et al., *J Clin Med* 2020^9^ | N=20 | Topical propranolol 1.5% gel vs. placebo, each applied twice daily | RCT (double-blind, placebo-controlled). Treatment: 8 weeks, each, followed by open-label period of 8 weeks | Epistaxis Severity Score | In propranolol gel and placebo gel groups, respectively, the median change (SD) in ESS was 2.03 (+/1.75) vs 0.35 (+/-0.68), which was a significant difference (*P*=0.004).  Significant improvement in hemoglobin and red cell units transfused |
| Peterson A. et al., *JAMA Otolaryngol Head Neck Surg* 2020^10^ | N=27 | Topical timolol 0.1% thermosensitive gel vs. placebo thermosensitive gel, each applied twice daily | RCT (double-blind, placebo-controlled).  Treatment: 2 months, each | Epistaxis Severity Score | In timolol gel and placebo gel groups, respectively, the median change (range) in ESS was 2.32 (0.22 to 5.97) vs 1.96 (−0.91 to 5.98), and 9 of 11 (82%) vs 9 of 12 (75%) participants experienced a clinically meaningful improvement in ESS |
| Riss D. et al. *Head Neck.* 2015^11^ | N=15 | Single dose of intranasal submucosal injection of bevacizumab or placebo | RCT (double-blind, placebo-controlled). Patients received a single intranasal submucosal injection of 100 mg of bevacizumab in 10 mL saline or placebo (10 mL saline) | The relation of the average daily post treatment epistaxis visual analog scale (VAS) score (range, 0–100) compared to the average daily pretreatment score in the month before the intervention (R=VAS-post/VAS-pre), for days 11-84. Patients recorded in a diary their daily epistaxis VAS scores ranging from 0 (best situation) to 100 (worst case). | Average daily VAS scores dropped from 18.8 (±16.5 SD) pretreatment to 13.4 (±11.6 SD) posttreatment in the bevacizumab group and from 20.5 (±13.4 SD) to 19.7 (±12.6 SD) in the placebo group, though the relation of the average daily posttreatment VAS score compared to the average daily pretreatment score did not show a statistically significant difference (*P*=.57) |
| Thompson K. et al., *Orphanet J Rare Dis* 2022^12^ | N=13 | Oral doxycycline hyclate 100 mg twice daily or placebo | RCT (double-blind, placebo-controlled crossover): Treatment: 6 months, each Washout period: 6 months | Weekly epistaxis duration change between treatment and placebo, compared to baseline | There was no significant difference in the change in weekly epistaxis duration (*P*=0.136) or frequency (*P*=0.261) between treatment and placebo |
| Whitehead K. et al., *JAMA* 2016^13^ | N=121 | Topical therapy with bevacizumab 1% (4 mg/d) or estriol 0.1% (0.4 mg/d) or  tranexamic acid 10% (40 mg/d) or placebo nasal sprays | RCT Phase II (double-blind, placebo controlled, stratified by epistaxis frequency) 4  treatment groups (bevacizumab 1% (4 mg/d), estriol 0.1% (0.4  mg/d), tranexamic acid 10% (40 mg/d), or placebo (0.9% saline) for 12 weeks. | Median weekly epistaxis frequency during weeks 5-12 for each patient | Epistaxis frequency was not significantly different between any of the active drug groups and the placebo group or between any of the therapeutic agents |
| Yaniv E. et al., *Laryngoscope* 2009^14^ | N=25 | Oral tamoxifen 20 mg once daily or placebo | RCT (double-blind, placebo controlled)  Treatment: 6 months, each | Frequency of epistaxis, duration of epistaxis, hemoglobin level | Epistaxis frequency was significantly lower with tamoxifen compared with placebo (*P*=.01), as was epistaxis “severity” (*P*=.049) at 6 months; no discussion of epistaxis duration in manuscript  There was no significant difference in hemoglobin between groups at 6 months |

**Supplementary Table 2.** Details of epistaxis diary design, epistaxis diary baseline characteristics, and diary-based outcomes from HHT randomized trials published between 2006-2025 utilizing an epistaxis diary.

| **Study** | **Sample Size & Intervention** | **Epistaxis Diary Type** | **Eligibility Requirements** | **Baseline Diary Measurements** | **Diary-Based Outcomes** | **Notes** |
| --- | --- | --- | --- | --- | --- | --- |
| Al-Samkari H. et al., *New Engl J Med* 2024^15^ | N=144 RCT, oral pomalidomide or placebo | Electronic (with paper option); frequency, duration, and intensity recorded | 3-month ESS ≥3.00 | Median monthly epistaxis duration at baseline 328 min (IQR 162-577 min)  Mean baseline ESS 5.0 | Median monthly epistaxis duration at 24 weeks: pomalidomide group 140 min, placebo group 244 min | Epistaxis duration was key secondary endpoint; difference between groups was not statistically significant, but post-hoc intensity-adjusted duration was (and magnitude of difference was much greater) |
| Dupuis-Girod S. et al., *JAMA* 2016^3^ | N=80 RCT, bevacizumab nasal spray or placebo nasal spray | Paper diary (“epistaxis grid”) used; only duration in minutes and frequency (up to 8/day) | >20 minutes of epistaxis per month prior to enrollment | Median monthly epistaxis duration:  Placebo 212 min (range 20-874) Bev 25 mg 200 min (21-1945) Bev 50 mg 141 min (33-754) Bev 75 mg 159 min (29-1739)  Mean monthly epistaxis duration:  Placebo 263 min (95% CI 158-368)  Bev 25 mg 286 min (83-488)  Bev 50 mg 229 min (128-330) Bev 75 mg 273 min (82-464) | Median monthly epistaxis duration:  Placebo 149 min (range 1-660) Bev 25 mg 164 min (3-1706) Bev 50 mg 141 min (8-1592) Bev 75 mg 147 min (8-970)  Mean monthly epistaxis duration:  Placebo 200 min (95% CI 109-292)  Bev 25 mg 259 min (82-436)  Bev 50 mg 244 min (82-406) Bev 75 mg 215 min (102-327) | Epistaxis duration was primary endpoint; difference between bevacizumab groups and placebo was not statistically significant |
| Dupuis-Girod S. et al., *J Intern Med* 2023^4^ | N=23 RCT, intravenous bevacizumab or placebo | Paper diary (“epistaxis grid”) used; only duration in minutes and frequency (up to 8/day) | No epistaxis-based eligibility criteria | Median monthly epistaxis duration:  Placebo 168.5 min (range 34-1467) Bevacizumab 310 min (range 46-2098) | Median monthly epistaxis duration:  Placebo 71 min (range 18-985) Bevacizumab 214 min (range 32-1163) | Dramatic imbalances in the groups at baseline (primary endpoint was hematologic, not epistaxis-based). No significant differences between baseline and post treatment seen |
| Gaillard S. et al., *J Thromb Haemost* 2014^5^ | N=135 Crossover RCT, oral tranexamic acid or placebo | Paper diary (“epistaxis grid”); only duration in minutes and frequency recorded | >28 episodes of epistaxis per month or >60 minutes of epistaxis per month | None | Median monthly epistaxis duration 122 min (IQR 69-231) in placebo period versus 106.2 min (IQR 52.6-212.8) in TXA period  Median monthly epistaxis frequency 22.1 (13.4-35.1) episodes/month in placebo versus 23.3 (11.2-36.5) episodes/month in TXA | Epistaxis duration was primary endpoint; improvement was statistically significant, corresponding to 17.3% difference between the two treatments |
| Geisthoff U. et al, *Thromb Res* 2014^6^ | N=22 Crossover RCT, oral tranexamic acid or placebo | Paper diary recording frequency, duration, intensity (on visual analog scale) | “Recurrent epistaxis” without further definition | None | Mean monthly epistaxis duration: TXA first group: 191 min on TXA period vs. 370 min on PBO period  PBO first group:  109 min/month on TXA period vs. 238 min/month on PBO period  Mean monthly epistaxis frequency:  21.6 episodes on TXA period vs. 30.0 on PBO period | Hemoglobin was primary endpoint (no significant difference)  Intensity-adjusted duration (called an “epistaxis score”) was secondary; this demonstrated an improvement of 54% on TXA versus placebo  Individual epistaxis parameters were analyzed post-hoc |
| Hermann R. et al., *Angiogenesis* 2024^7^ | N=60 RCT, oral nintedanib or placebo | Paper diary (“epistaxis grid”); only duration in minutes and frequency recorded  Protocol makes note of epistaxis grid being able to be filled out “online”, but no other discussion of nature of electronic diary is present | ESS ≥4.00 | Median monthly epistaxis duration:  Placebo 258 min (IQR 147-350) Nintedanib 264 min (IQR 138-436)  Mean monthly epistaxis duration (SD): Placebo 274 (154) min  Nintedanib 314 min (227)  Median monthly epistaxis frequency: Placebo 29 episodes (IQR 17-43) Nintedanib 40 episodes (19-56)  Mean monthly epistaxis frequency (SD): Placebo 33 episodes (20) Nintedanib 40 episodes (25) | At 8 weeks of follow up (W13-W20):  Median monthly epistaxis duration:  Placebo 156 minutes (IQR 100-201) Nintedanib 82 minutes (IQR 13-219)  Mean monthly epistaxis duration (SD): Placebo 172 (105) min Nintedanib 155 (214)  Median monthly epistaxis frequency: Placebo 19 episodes (IQR 14-37) Nintedanib 11 episodes (6-45)  Mean monthly epistaxis frequency (SD): Placebo 27 episodes (20) Nintedanib 26 episodes (27) | Epistaxis response (50% reduction in duration at last 8 weeks of treatment, W5-W12) was primary endpoint; this was not significantly different between groups  At follow up (W13-W20), there was a significant improvement in epistaxis duration and epistaxis frequency |
| McWilliams J. et al., *J Thromb Haemost* 2022^8^ | N=22 Crossover RCT, oral doxycycline or placebo | Paper diary recording frequency, duration, and intensity | ESS≥4.00, ≥3 episodes of epistaxis per week and ≥15 minutes of epistaxis per week | Mean monthly epistaxis duration (SD) 345 min (383.2)  Mean monthly epistaxis frequency (SD): 40 (29)  Mean (SD) ESS 6.0 (1.4) | Reduction in mean (SD) monthly epistaxis duration between groups -262 min (701)  Reduction in mean (SD) monthly epistaxis frequency between groups -11.2 (33.6) | None of the primary (or secondary) endpoints was statistically significantly improved  Most of the measured difference in epistaxis duration, and about half of the difference in epistaxis frequency, between the two groups came from worsening of the placebo group from baseline rather than improvement of the doxycycline group |
| Riss D. et al, *Head Neck* 2015^11^ | N=15 RCT, single dose of intranasal submucosal bevacizumab or placebo | Paper diary recording only “epistaxis visual analog score (VAS)” (single measurement) | ≥2 episodes of epistaxis per week | Daily epistaxis visual analog scale was recorded for 1 month prior to intervention | Average daily VAS scores dropped from 18.8 (±16.5 SD) pretreatment to 13.4 (±11.6 SD) posttreatment in the bevacizumab group and from 20.5 (±13.4 SD) to 19.7 (±12.6 SD) in the placebo group | The difference was not statistically significant |
| Thompson K. et al, *Orphanet J Rare Dis* 2022^12^ | N=13 Crossover RCT, oral doxycycline or placebo | Paper diary recording frequency, duration, and intensity | ≥15 minutes of epistaxis per week | Mean monthly epistaxis duration 136 minutes (range, 72-1096)  Median (range) baseline ESS 4.96 (2.43-7.28) | Mean weekly epistaxis duration increased 1 minute after doxycycline treatment and decreased 14 minutes after placebo treatment  Mean (SD) weekly epistaxis frequency 7.5 (3.6) at beginning of doxycycline, 6.8 (3.3) at end; mean (SD) weekly epistaxis frequency 8.9 (4.1) at beginning of placebo, 6.2 (3.3) at end | No improvement in epistaxis duration or frequency on doxycycline |
| Whitehead K. et al., *JAMA* 2016^13^ | N=121 RCT, topical nasal spray (bevacizumab, estriol, tranexamic acid, or placebo) | Paper diary recording frequency and duration only | ESS ≥3.00; experienced epistaxis lasting at least 1 minute, occurring at least once weekly, and being stable in the previous 8 weeks | None | Median weekly epistaxis frequency:  Bevacizumab 7.0 (IQR, 4.5-10.5)  Estriol 8.0 (IQR, 4.0-12.0)  Tranexamic acid 7.5 (IQR, 3.0-11.0)  Placebo 8.0 (IQR, 3.0-14.0)  Monthly median (IQR) epistaxis duration by group:  Placebo: 184 min (40-336)  Bevacizumab: 94 min (52-160)  Estriol: 146 (28-280)  TXA: 178 (54-350) | Epistaxis frequency was the primary endpoint, and was not significantly different between any of the active groups and placebo group or between any active agent |
| Yaniv E. et al., *Laryngoscope* 2009^14^ | N=25 Oral tamoxifen or placebo | Paper diary recording epistaxis frequency, duration and “severity” | Grade 2 or 3 epistaxis according to the standardized protocol of Bergler et al. 2002 (PMID 1191682) | None | No specific information given in the publication: “By the end of treatment, there was no change in the epistaxis in the placebo group. There was a significant difference between the groups at the 6-month follow-up in both frequency (*P*=.01) and severity (*P*=.049) of epistaxis.” | Inadequate reporting in publication; primary and secondary endpoints not specified; no protocol available with publication  Also evaluated telangiectasia severity by nasal endoscopy before and after intervention |

**EXTERNAL REVIEW OF REPORT: PROCESS AND RESULTS**

The draft ICR manuscript and supplement were reviewed by topic experts and practicing physicians caring for HHT patients who were not involved in the development of the report who represented many different specialties and subspecialties and who were from many different countries around the globe. External reviewers were asked to read the ICR manuscript and supplement and then to complete an online, fully anonymous external reviewer survey instrument. This instrument, hosted by Survey Monkey, was used to collect and tabulate responses from external reviewers. Invitations were sent out to 23 potential external reviewers from 9 different countries (Australia, Canada, Finland, Netherlands, Saudi Arabia, South Africa, New Zealand, United Arab Emirates, and United States), of whom 19 accepted the invitation, none declined the invitation, and 4 did not respond to the invitation. Of the 19 accepting the invitation, the review was completed and responses to the anonymous external reviewer questionnaire were received by 15 reviewers.

**Responses to Required Yes/No Questions from External Reviewers:**

| Question | Total responses, n (%) | Yes, n (%) | No, n (%) | No opinion, n (%) |
| --- | --- | --- | --- | --- |
| Do you believe this report addresses the most important gaps and lack of standardization in terminology, definitions, and outcome criteria for HHT-associated bleeding? | 15 (100) | 14 (93) | 1 (7) | 0 (0) |
| Does this report appropriately balance standardization of terminology, definitions, and outcome criteria with the flexibility required to design HHT clinical trials? | 15 (100) | 15 (100) | 0 (0) | 0 (0) |
| Do you believe the systematic literature review was adequately comprehensive and robust for the topics of HHT-associated epistaxis and gastrointestinal bleeding? | 15 (100) | 15 (100) | 0 (0) | 0 (0) |
| **I would recommend this report for use in clinical study design and by regulatory agencies evaluating therapeutic development for HHT-associated bleeding.** | **15 (100)** | **15 (100)** | **0 (0)** | **0 (0)** |

**Required Rating of Overall ICR Quality:** Reviewers were asked to rate the overall quality of the ICR on a scale of 1 to 5, in which 5 was high quality (defined as impactful, clear, relevant to HHT clinical study design, and rigorous methodology) and 1 was low quality (defined as low impact, unclear, lack of relevance to HHT clinical study design and weak methodology).

| **Question** | **1 (low), n (%)** | **2, n (%)** | **3, n (%)** | **4, n (%)** | **5 (high), n (%)** | **Weighted average** | **Total responses, n (%)** |
| --- | --- | --- | --- | --- | --- | --- | --- |
| Rate the overall quality of this consensus report. | 0 (0) | 0 (0) | 0 (0) | 2 (13) | 13 (87) | **4.87/5.00** | 15 (100) |

**Input Regarding Literature Review and Essential References:** External reviewers were additionally asked if they believed any pertinent references were not included in the manuscript or supplementary appendix, to enter the pertinent missing references along with the topic/specific evidence covered by the reference. All of the external reviewers responding to this optional question noted that they felt no essential references were missing and did not specify any specific missing references.

**Input Regarding Future Directions for Standardization in HHT-associated bleeding:** External reviewers were asked to suggest future directions for standardization in HHT-associated bleeding. Reviewers commented that this ICR is an excellent foundation for global collaboration. External reviewers recommended the following:

- Additional assessment of heavy menstrual bleeding in HHT clinical trials
- The development of HHT-specific electronic apps to calculate disease severity
- Greater investigation of NOSE HHT regarding its optimal use and place in HHT clinical trials (highlighting that it has many similarities with HRQoL instruments and that patients who under-report symptoms may have low scores despite being objectively severe, i.e. transfusion-dependent)
- Additional assessment of the impact of diet or supplements on bleeding outcomes
- One reviewer commented on the need for a universal bleeding scale in HHT that characterizes overall bleeding from all sites

**Input Regarding Additional Comments or Concerns Regarding the Report or Any of the Recommendations:** External reviewers were asked to state any additional comments or concerns regarding the ICR, if they had any. Several reviewers were gave congratulatory and other laudatory statements regarding the completion of the effort and its high quality. Reviewers additionally commented:

- The Hematologic Support Score and Hematologic Impact Score were very welcomed additions to the HHT clinical trial endpoint and clinical assessment space
- Some reviewers supported universal use of the ESS only (over NOSE HHT) due to its simplicity and the fact that it has been more consistently used in prospective therapeutic trials, while others commented on challenges with the instrument (all of which are discussed on page 16 of this Supplementary Appendix).

**DETAILS OF VALIDATED EPISTAXIS SEVERITY INSTRUMENTS DISCUSSED IN REPORT**

**Epistaxis Severity Score**

The epistaxis severity score (ESS) is a 6-question epistaxis severity instrument used specifically to evaluate epistaxis severity in HHT.^15^ A score of 0.00-1.00 is considered minimal epistaxis, 1.01-4.00 is considered mild epistaxis, 4.01 to 7.00 is considered moderate epistaxis, and 7.01-10.00 is considered severe epistaxis. The ESS is assessed over a prespecified reference time interval, usually the past 1-3 months. The ESS was developed in the United States in English.

This instrument utilizes six factors identified as independent predictors of self-described epistaxis severity, each weighed by different coefficients derived from a multivariable model. The minimal clinically important difference of the ESS is 0.71 points.^16^

An advantage of the ESS is that it may be assessed rapidly in the clinic (though it requires a calculator to score as it employs complex arithmetic). It is validated and has been used in all major HHT-associated bleeding clinical trials over the past several years. However, it has a number of drawbacks, which are important to consider when considering its use:

1. It includes a question about blood transfusion specifically due to epistaxis, which in practice can be difficult to adjudicate as gastrointestinal blood loss and epistaxis blood loss often coexist.
2. It asks only about red cell transfusion, not intravenous iron infusion, and proper iron deficiency management in HHT means that even most of the patients at the severe end of the bleeding spectrum can maintain normal or near-normal hemoglobin values with very aggressive IV iron infusion alone, without requiring red cell transfusion.
3. It asks a question about healthcare utilization, which is imprecise unless the definition of healthcare utilization is prespecified in advance. Moreover, many patients with HHT are hesitant to utilize emergency room services due to the painful and sometimes drastic measures employed to stop nosebleeds.
4. Relatively modest changes in reporting by the patient can result in notable shifts in scoring.
5. Despite the high test-retest reliability of the ESS, a recent study found relatively low internal consistency with the instrument (suggesting that it may measure multiple unrelated concepts, such as gastrointestinal blood loss in addition to epistaxis severity).^17^

At the time of writing, a free online ESS calculator is hosted at <https://curehht.org/resource/epistaxis-severity-score/>.

Epistaxis Severity Score Instrument^15^


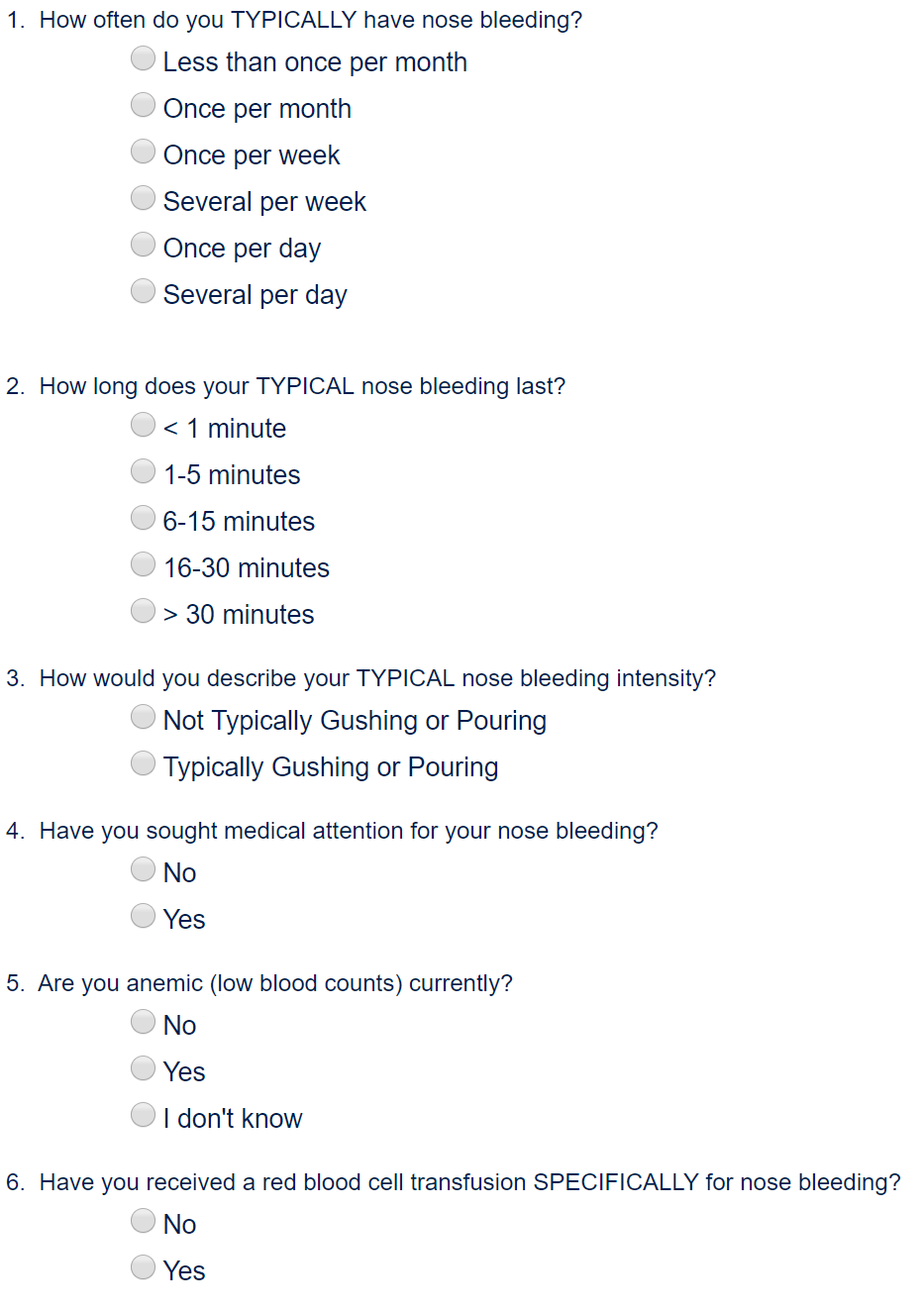


**Nasal Outcome Score for Epistaxis in Hereditary Hemorrhagic Telangiectasia**

The Nasal Outcome Score for Epistaxis in Hereditary Hemorrhagic Telangiectasia (NOSE HHT) is a 29-item epistaxis severity instrument scored on a 4-point scale, in which 0.00-1.00 is mild epistaxis, 1.01-2.00 is moderate epistaxis, and 2.01 to 4.00 is severe epistaxis.^18^ The reference time interval for the instrument is always 2 weeks. NOSE HHT was developed in the United States in English. It is scored by averaging the score of all answered questions (the answer to each question is worth 0-4 points). The minimal clinically important difference for NOSE HHT is 0.46 points. While NOSE HHT has been validated, at the time of writing it has yet to be used in a large HHT randomized clinical trial. Advantages include the breadth of questions asked, which cover important aspects of the impact of epistaxis on patients missed by the ESS (such as nocturnal bleeds, bleeding impacting social activities or work, etc.). A primary disadvantage is the length of the instrument relative to the 6-question ESS. A free online calculator for NOSE-HHT is available (<https://outcomesresearch.github.io/nose-hht/>).

Nasal Outcome Score for Epistaxis in Hereditary Hemorrhagic Telangiectasia Instrument^18^


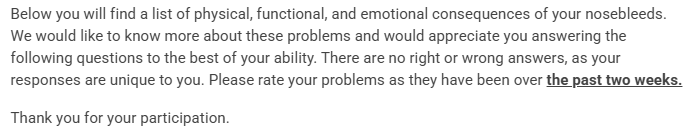

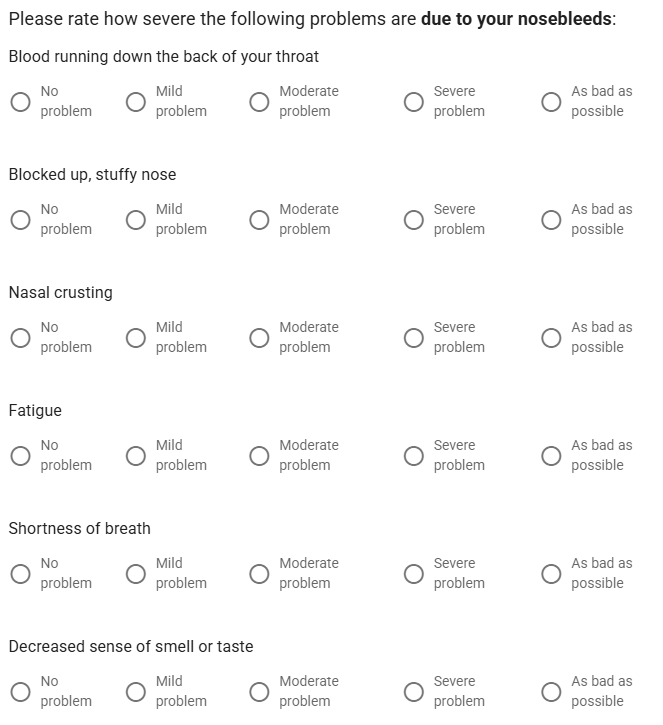


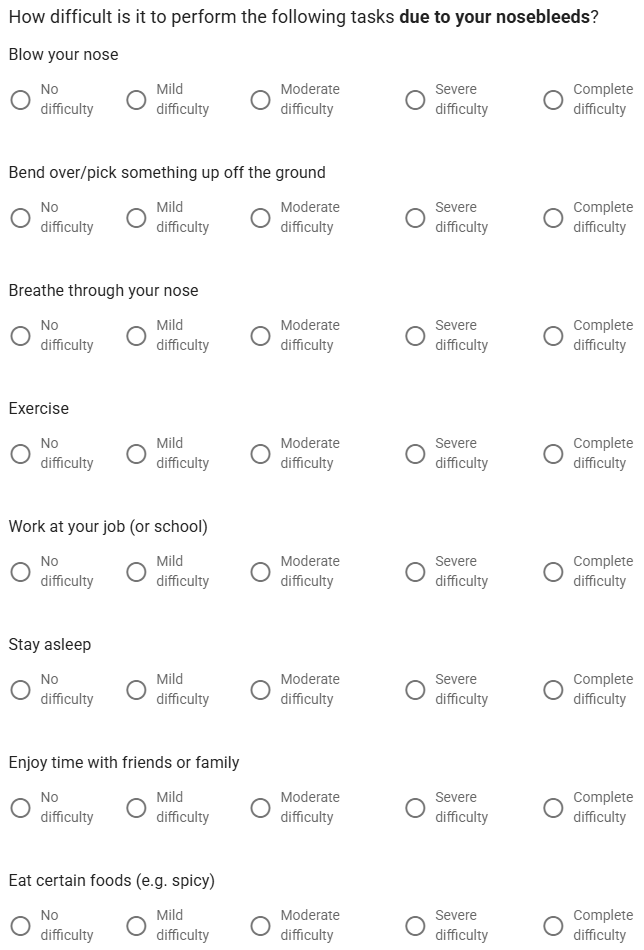


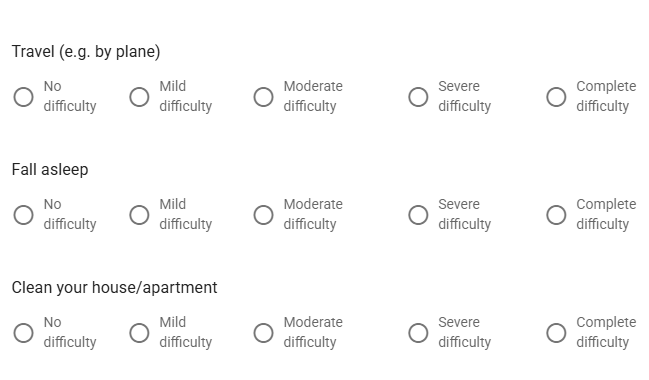


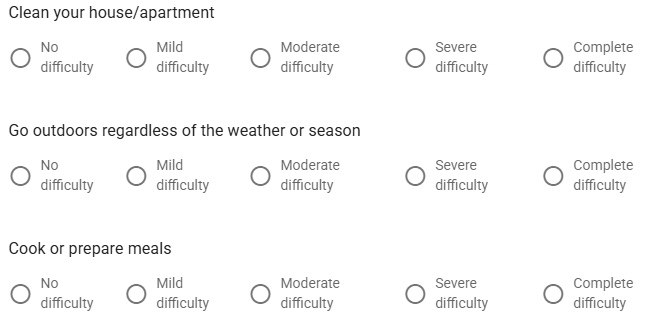


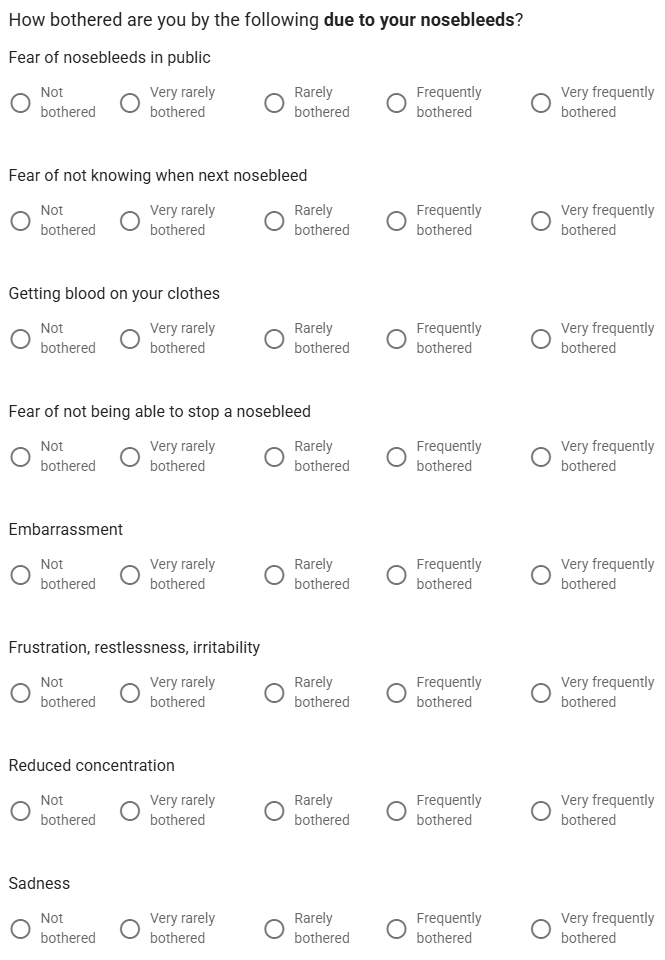


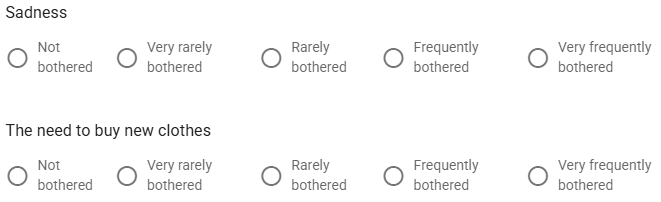


**JUSTIFICATION FOR NUMERICAL THRESHOLDS IN BLEEDING SEVERITY CLASSIFICATION**

**Domain 1: Objective Epistaxis Severity Measurement**

The thresholds for ESS and NOSE HHT directly correspond to the definitions of mild, moderate, and severe epistaxis defined by the scoring systems for these validated epistaxis severity instruments.

The epistaxis diary criteria for moderate severity in domain 1 (monthly frequency of ≥20 episodes plus monthly duration of ≥80 cumulative minutes) was adapted from the eligibility criteria of the VAD044002 trial,^19^ where it was put in place as the threshold for the definition of moderate epistaxis severity alongside an ESS of ≥4. The expert panel considered alternatives, including modifying the numerical thresholds in this definition and separate definitions incorporating only frequency or duration, rather than requiring both. Ultimately, the aforementioned definition was adopted as (1) it was felt to be adequately rigorous, requiring over an hour of epistaxis in a four-week period plus an average of 5 or more episodes per week; (2) it had already been utilized successfully in a successful randomized, placebo-controlled trial; (3) in order for it to be utilized in the VAD044002 trial as it was, it underwent scrutiny as a definition of moderate epistaxis severity in HHT by both the U.S. Food and Drug Administration and European Medicines Agency and passed in both cases; and (4) utilization of only epistaxis frequency or duration criteria was felt to be problematic; a combination of both a frequency and duration threshold was felt to be optimal to ensure that patients with very minimal nosebleed duration (e.g., <1 minute but 1-2 episodes per day) or with one or two nosebleeds per month that happen to last a very long time do not meet moderate or severe criteria as these patients would be considered major outliers in the setting of a clinical trial.

The epistaxis diary criteria for severe severity in domain 1 (monthly frequency of ≥30 episodes plus monthly duration of ≥200 cumulative minutes) was agreed upon by the expert panel following a thorough evaluation of the accumulated epistaxis diary data from the literature search (**Supplemental Table 2**), as well as the recognition that this definition requires patients to have nosebleeding on average at least once daily *and* accumulate over 50 minutes of bleeding per week, on average, which was felt to be severe without any reasonable doubt. Similarly, a definition requiring both frequency and duration thresholds was recognized to minimize variability in enrolled clinical trial patients.

**Domain 2: Hematologic Support Requirements to Maintain Normal Hemoglobin and Normal Iron Stores**

Defining mild severity in domain 2 as maintenance of a normal hemoglobin and normal iron stores with dietary iron or oral iron supplementation alone was non-controversial and in line with the mild gastrointestinal bleeding severity in the Second International HHT Guidelines.

Also in line with the Second International HHT Guidelines was the requirement for parenteral iron as the threshold for defining moderate severity. A requirement for parenteral iron in patients with HHT, however, can range from intermittent infusions of small amounts of iron (e.g., 100-250 mg elemental iron) to regular infusions of high-dose iron (500-1000 mg elemental iron or more). Defining moderate as <2000 mg infused in the preceding 12 months and severe as 2000 mg or more transfused in the preceding 12 months was determined on the basis of (1) the recognition that 2000 mg infused generally ranges between 2-10 separate iron infusion episodes depending on the type of iron used, which is significant to have to do on a yearly basis, and (2) a recognition of the rapidity of total body iron store depletion in a patient losing up to 2000 mg of IV iron every year. Because the average adult female has total body extra-erythron iron stores of approximately 1000 mg and an average adult male has total body extra-erythron iron stores of approximately 1500 mg, a requirement of 2000 mg of parenteral iron on a yearly basis defines a patient who bleeds out their entire stores of iron every 6-9 months, which was agreed upon by the expert panel as a sufficiently rapid rate of iron loss to define 2000 mg or more as severe bleeding. Additionally, patients with moderate bleeding may have a small number of acute bleeding episodes over long spans of time (years) that each may require a single episode of red cell transfusion, highlighting the need to include an RUE-based definition in the moderate severity range (which equals the IV iron only threshold of up to 2000 mg, as up to 2000 mg IV iron equals up to 8 RUEs) rather than just determining that a requirement for any red cell transfusion means the patient falls in the severe category.

Defining a requirement for red cell transfusion to maintain an acceptable (safe) hemoglobin as the special category of very severe was agreed upon by the expert panel given the uniquely challenging clinical circumstances of this group. These individuals usually require visits to the hospital or an infusion suite once to twice weekly and experience a very high burden of both anemia and iron deficiency symptoms, of healthcare utilization, of overall medicalization of their lives, and of risk for complications of red cell transfusions (including alloimmunization, which ranges from 15-37% in the HHT population.^20,21^ The exact prevalence of this very severe class is not known (but is a topic of future research) but is thought to represent fewer than 5-10% of patients with HHT who receive proper intravenous iron management.

**RED-CELL UNIT EQUIVALENT (RUE) BACKGROUND**

The approximate quantity of elemental iron present in a unit of packed red cells administered to adult patients is 250 mg.^22,23^ There is some variation in this number from unit to unit, and from country to country; however, on average, the quantity approximates 250 mg per unit. Because most patients with HHT have a normal functioning bone marrow, and iron is the limiting factor in erythropoiesis in iron deficiency anemia, for the vast majority patients with HHT, intravenous iron infusion is essentially equivalent to a time-delayed red cell transfusion. Therefore, the red-cell unit equivalent (RUE) allows for the easy combination of two otherwise very numerically disparate hematologic support modalities (red cell units transfused, which is measured with 1-2 digits, and milligrams of elemental iron infused, which is measured with 3-4 digits) in a biochemically sound manner. One unit of red cells transfused equals 1 RUE, and every 250 mg of elemental iron infused equals 1 RUE (the total elemental iron given in milligrams is simply divided by 250 to convert the iron infused into RUEs). This allows for logical severity stratification (see Table 1 in the main manuscript) as well as the use of composite hematologic endpoints in HHT clinical trials (see Hematologic Measures and Endpoints in the main manuscript), which avoids the significant pitfalls of use of single/solitary hematologic endpoints (see below).

The expert panel acknowledges both the existing evidence and convention that allows the conversion between milligrams of elemental iron and RUEs, but also recognizes that there may be circumstances (as of now unknown) in which the conversion is less biologically valid. For this reason, as noted in the main manuscript, the expert panel recommends the continued use of flawed solitary hematologic endpoints in addition to composite hematologic endpoints as non-key secondary or exploratory endpoints in clinical trials, which will ultimately allow for additional study to identify potential circumstances in which the conversion could be less valid.

**PITFALLS OF SINGLE/SOLITARY HEMATOLOGIC ENDPOINTS IN HHT TRIALS**

Below are three example scenarios illustrating the danger of using a solitary hematologic endpoint (in this case, hemoglobin, although the same pitfalls can occur with use of red cell transfusions alone or milligrams of intravenous iron infused alone) and how these pitfalls are avoided by using a composite hematologic endpoint, such as the Hematologic Support Score, or HSS and Hematologic Impact Score or HIS (see Figure 1 in the main manuscript for more explanation on composite hematologic endpoints).

|  | Pretreatment (Prior to Trial Enrollment) | | | | On Treatment (Following Trial Enrollment) | | | | Difference with Treatment | | |
| --- | --- | --- | --- | --- | --- | --- | --- | --- | --- | --- | --- |
| Pt # | Units RBCs | Mg Fe Infused | Hgb | 6-Mo PreTx HSS | Units RBCs | Mg Fe Infused | Hgb | 6-Mo PostTx HSS | Hemoglobin Relative Change | HSS Relative Change | HIS Absolute Change |
| 1 | 0 | 1000 | 14.5 | 4 | 0 | 250 | 14.1 | 1 | 3% worse | 75% better | +2.6 RUEs |
| 2 | 0 | 500 | 7.9 | 2 | 1 | 1500 | 12.8 | 7 | 113% better | 250% worse | -0.1 RUEs |
| 3 | 12 | 5000 | 7.6 | 32 | 6 | 3000 | 7.1 | 18 | 7% worse | 37% better | +13.5 RUEs |

Patient 1 illustrates marked success in reducing bleeding with treatment, as noted by a 75% improvement in hematologic support requirements while maintaining a similar hemoglobin; this is completely missed if hemoglobin alone is the outcome.

Patient 2 illustrates how hemoglobin alone would suggest that the patient improved in terms of bleeding, with a drastic improvement (~5 grams), but the HSS and HIS confirm that this patient’s bleeding did not improve at all, and that what happened here was that he received proper hematologic support with IV iron and red cell transfusion during the trial (presumably due to protocol-required infusions) that he was not receiving before enrolling on the trial.

Patient 3 illustrates a patient with very severe transfusion-dependent anemia. Her hemoglobin is slightly lower post-treatment, but as demonstrated by her HSS and HIS, the treatment has clearly meaningfully improved her bleeding given the improvement in hematologic support requirements.

In every circumstance, hemoglobin alone is misleading, and only a suitable composite endpoint gave the complete picture of the impact of treatment, or lack thereof, on the patient’s hematologic status.

**HHT-SPECIFIC HEALTH-RELATED QUALITY OF LIFE INSTRUMENTS**

**HHT-Specific Quality of Life Scale (HHT-QoL)**

The HHT-QoL Scale is a simple, 4-question HHT-specific HRQoL instrument developed with comprehensive data (including HHT-QoL scale, 3 PROMIS measures, and self-report of HHT disease severity) from 290 patients with HHT.^24^ The instrument is scored between 0 (no impact of HHT on HRQoL) to 16 (worst impact of HHT on HRQoL). The HHT-QoL Scale was developed in the United States in English and was utilized in the NIH-funded PATH-HHT study (NCT03910244) of pomalidomide,^1^ in which it was a key secondary endpoint and from which its initial validation (144 patients) is anticipated; it is also being utilized in the ongoing NIH-funded TrUST-HHT study (NCT04404881) evaluating systemic bevacizumab in HHT.

HHT-Specific Quality of Life Scale Instrument^24^

Please answer all questions:

1. How often in the past 4 weeks has an activity for your work, school, or regularly scheduled commitments been ***interrupted by a nose bleed***?

0=Never

1=Rarely

2=Sometimes

3=Often

4=Always

2. How often in the past 4 weeks has an activity with your partner, family, or friends been ***interrupted by a nose bleed?***

0=Never

1=Rarely

2=Sometimes

3=Often

4=Always

3. How often in the past 4 weeks have you ***avoided social activities*** because you were ***worried about having a nose bleed***?

0=Never

1=Rarely

2=Sometimes

3=Often

4=Always

4. How often in the past 4 weeks have you ***had to miss*** your work, school, or regularly scheduled commitments because of ***HHT-related problems other than nosebleeds?***

0=Never

1=Rarely

2=Sometimes

3=Often

4=Always

**Scoring:**

A total score is calculated by summing the items (each scored from 0-4). The total score ranges from 0 (no limitations) to 16 (severe limitations). If more than one item is not answered, the total score will not be calculated. If one item is missing then the total score is calculated as the sum of the 3 items multiplied by 1.33.

**Quality of Life Questionnaire in HHT**

The Quality of Life Questionnaire in HHT (QoL-HHT) is a more comprehensive 24-item HHT-specific QoL instrument developed in a calibration sample of 415 patients with HHT and validated in a separate validation sample of 228 patients with HHT.^25^ The QoL-HHT was developed in France in French. The instrument measures 6 domains of HRQoL: physical limitations, social relationships, concern about bleeding, relationship with the medical profession, experience of symptoms, and concern about the evolution of the disease. The original French version of the QoL-HHT is provided below, as per the instrument’s published manuscript. The English version of the QoL-HHT is currently undergoing validation, and this validation along with the formal English translation is expected to be published in the coming months. As described in the main manuscript, investigators wishing to use this instrument in another language should have the instrument translated by a professional translation service according to the standards of the industry.

Quality of Life Questionnaire in HHT Instrument^25^


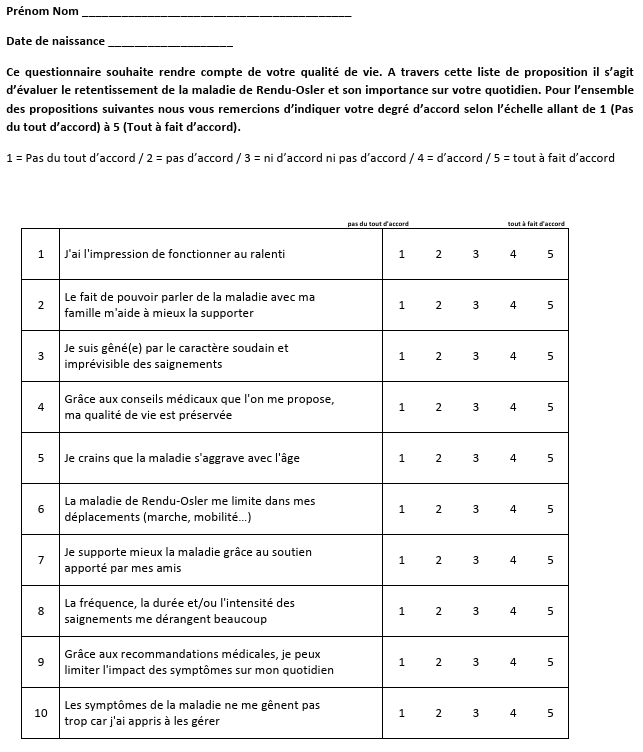


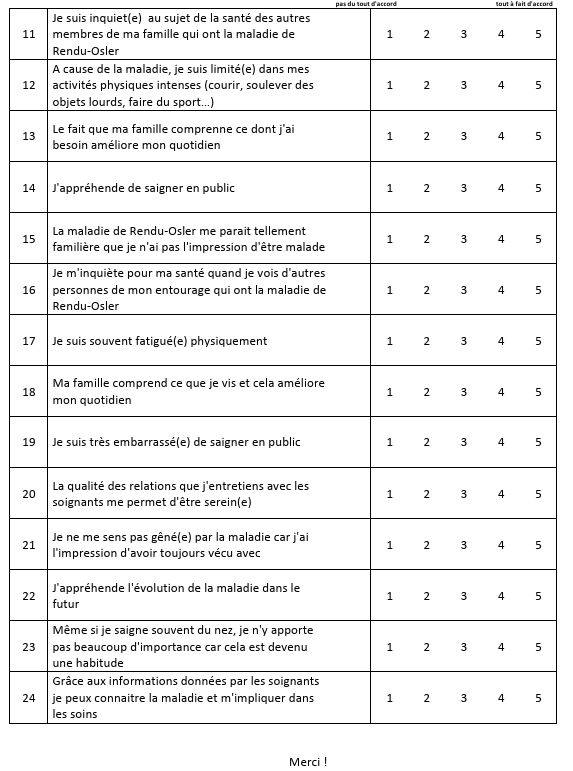


Scoring: Each dimension of the QoL-HHT is scored from 0-100, with a higher number indicating a better quality of life.

**ILLUSTRATIVE EXAMPLES OF EPISTAXIS RESPONSE CRITERIA**

The below figure illustrates 3 example patients on a clinical trial utilizing the epistaxis response criteria described in Table 3 in the main manuscript. In the case of the example, the clinical trial involves 6 months of treatment with a hypothetical drug and utilizes a primary endpoint of intensity-adjusted epistaxis duration (proportion of participants with an epistaxis response [ER] or optimal epistaxis response [OER] at month 6).


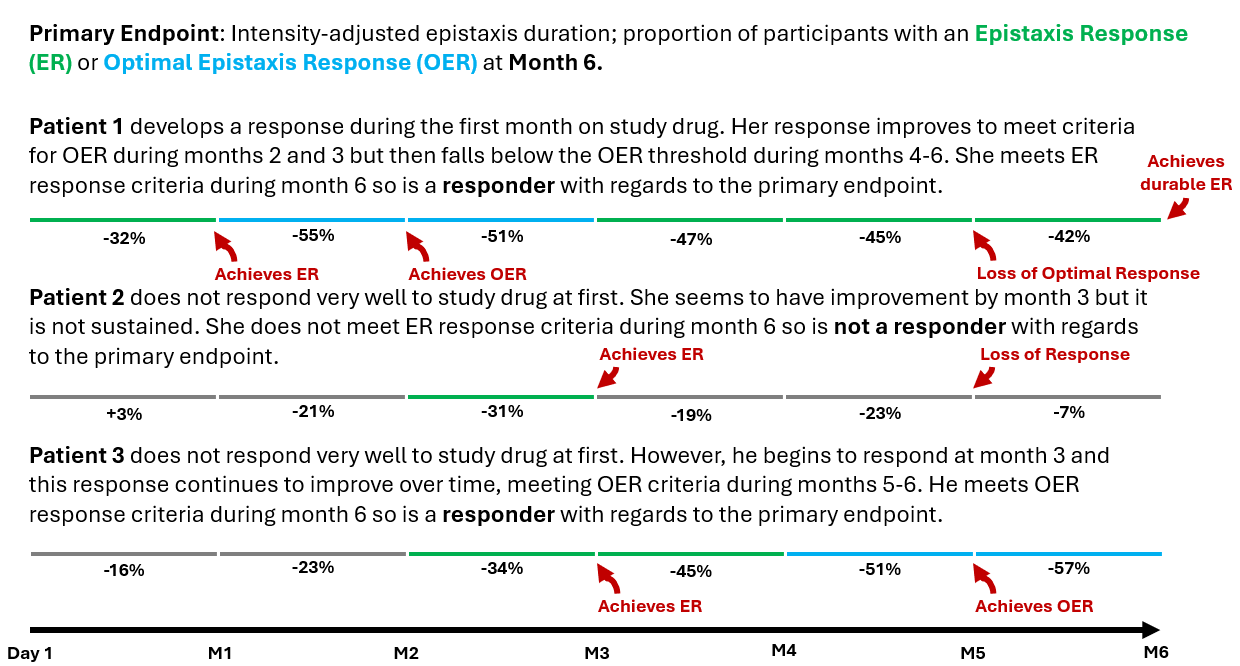


**SUPPLEMENTARY APPENDIX REFERENCES**

1. Al-Samkari H, Kasthuri RS, Iyer VN, et al. Pomalidomide for Epistaxis in Hereditary Hemorrhagic Telangiectasia. *N Engl J Med* 2024; **391**(11): 1015-27.

2. Boyer H, Fernandes P, Le C, Yueh B. Prospective randomized trial of sclerotherapy vs standard treatment for epistaxis due to hereditary hemorrhagic telangiectasia. *Int Forum Allergy Rhinol* 2015; **5**(5): 435-40.

3. Dupuis-Girod S, Ambrun A, Decullier E, et al. Effect of Bevacizumab Nasal Spray on Epistaxis Duration in Hereditary Hemorrhagic Telangectasia: A Randomized Clinical Trial. *JAMA* 2016; **316**(9): 934-42.

4. Dupuis-Girod S, Riviere S, Lavigne C, et al. Efficacy and safety of intravenous bevacizumab on severe bleeding associated with hemorrhagic hereditary telangiectasia: A national, randomized multicenter trial. *J Intern Med* 2023; **294**(6): 761-74.

5. Gaillard S, Dupuis-Girod S, Boutitie F, et al. Tranexamic acid for epistaxis in hereditary hemorrhagic telangiectasia patients: a European cross-over controlled trial in a rare disease. *J Thromb Haemost* 2014; **12**(9): 1494-502.

6. Geisthoff UW, Seyfert UT, Kubler M, Bieg B, Plinkert PK, Konig J. Treatment of epistaxis in hereditary hemorrhagic telangiectasia with tranexamic acid - a double-blind placebo-controlled cross-over phase IIIB study. *Thromb Res* 2014; **134**(3): 565-71.

7. Hermann R, Grobost V, Le-Guillou X, et al. Effect of oral nintedanib vs placebo on epistaxis in hereditary hemorrhagic telangiectasia: the EPICURE multicenter randomized double-blind trial. *Angiogenesis* 2024; **28**(1): 9.

8. McWilliams JP, Majumdar S, Kim GH, et al. North American Study for the Treatment of Recurrent Epistaxis with Doxycycline: The NOSTRIL trial. *J Thromb Haemost* 2022; **20**(5): 1115-25.

9. Mei-Zahav M, Gendler Y, Bruckheimer E, et al. Topical Propranolol Improves Epistaxis Control in Hereditary Hemorrhagic Telangiectasia (HHT): A Randomized Double-Blind Placebo-Controlled Trial. *Journal of clinical medicine* 2020; **9**(10).

10. Peterson AM, Lee JJ, Kallogjeri D, Schneider JS, Chakinala MM, Piccirillo JF. Efficacy of Timolol in a Novel Intranasal Thermosensitive Gel for Hereditary Hemorrhagic Telangiectasia-Associated Epistaxis: A Randomized Clinical Trial. *JAMA Otolaryngol Head Neck Surg* 2020; **146**(11): 1006-14.

11. Riss D, Burian M, Wolf A, Kranebitter V, Kaider A, Arnoldner C. Intranasal submucosal bevacizumab for epistaxis in hereditary hemorrhagic telangiectasia: a double-blind, randomized, placebo-controlled trial. *Head Neck* 2015; **37**(6): 783-7.

12. Thompson KP, Sykes J, Chandakkar P, et al. Randomized, double-blind, placebo-controlled, crossover trial of oral doxycycline for epistaxis in hereditary hemorrhagic telangiectasia. *Orphanet J Rare Dis* 2022; **17**(1): 405.

13. Whitehead KJ, Sautter NB, McWilliams JP, et al. Effect of Topical Intranasal Therapy on Epistaxis Frequency in Patients With Hereditary Hemorrhagic Telangiectasia: A Randomized Clinical Trial. *JAMA* 2016; **316**(9): 943-51.

14. Yaniv E, Preis M, Hadar T, Shvero J, Haddad M. Antiestrogen therapy for hereditary hemorrhagic telangiectasia: a double-blind placebo-controlled clinical trial. *Laryngoscope* 2009; **119**(2): 284-8.

15. Hoag JB, Terry P, Mitchell S, Reh D, Merlo CA. An epistaxis severity score for hereditary hemorrhagic telangiectasia. *Laryngoscope* 2010; **120**(4): 838-43.

16. Yin LX, Reh DD, Hoag JB, et al. The minimal important difference of the epistaxis severity score in hereditary hemorrhagic telangiectasia. *Laryngoscope* 2016; **126**(5): 1029-32.

17. Gong AJ, Bolsegui ML, Lee EE, Mathai SC, Weiss CR. Assessing the Psychometric Validity of the Epistaxis Severity Score: Internal Consistency and Test-Retest Reliability. *Am J Rhinol Allergy* 2024; **38**(1): 38-46.

18. Peterson AM, Kallogjeri D, Spitznagel E, Chakinala MM, Schneider JS, Piccirillo JF. Development and Validation of the Nasal Outcome Score for Epistaxis in Hereditary Hemorrhagic Telangiectasia (NOSE HHT). *JAMA Otolaryngol Head Neck Surg* 2020; **146**(11): 999-1005.

19. Al-Samkari H, Hessels J, Riera-Mestre A, et al. A Randomized, Placebo-Controlled, Multicenter Proof-of-Concept (POC) Study to Assess the Safety and Efficacy of the Novel Allosteric AKT Inhibitor, VAD044, in Adults with Hereditary Hemorrhagic Telangiectasia (HHT). *Blood* 2024; **144**(Suppl 1): 553.

20. Grudzinski A, Tse B, Ombao R, Faughnan ME, Pavenski K. Red blood cell alloimmunization in transfused patients with hereditary hemorrhagic telangiectasia: A single centre retrospective study. *Transfus Apher Sci* 2024; **63**(6): 104019.

21. Zheng Y, Pollak J, Henderson K, Hendrickson JE, Tormey CA. A novel association between high red blood cell alloimmunization rates and hereditary hemorrhagic telangiectasia. *Transfusion (Paris)* 2018; **58**(3): 775-80.

22. Bernhart FW, Skeggs L. The Iron Content of Crystalline Human Hemoglobin. *J Biol Chem* 1942; **147**: 19-.

23. Elgailani IEH, Alsakka MM. Determination of Iron Content in Different Hemoglobin Samples from Some Patients by UV-Visible Spectrophotometer. *Advances in Analytical Chemistry* 2016; **6**(2): 35-40.

24. Kasthuri RS, Chaturvedi S, Thomas S, et al. Development and performance of a hereditary hemorrhagic telangiectasia-specific quality-of-life instrument. *Blood advances* 2022; **6**(14): 4301-9.

25. Le TTT, Martinent G, Dupuis-Girod S, et al. Development and validation of a quality of life measurement scale specific to hereditary hemorrhagic telangiectasia: the QoL-HHT. *Orphanet J Rare Dis* 2022; **17**(1): 281.
